# ImmuneLENS characterizes systemic immune dysregulation in aging and cancer

**DOI:** 10.1038/s41588-025-02086-5

**Published:** 2025-02-18

**Authors:** Robert Bentham, Thomas P. Jones, James R. M. Black, Carlos Martinez-Ruiz, Michelle Dietzen, Maria Litovchenko, Kerstin Thol, Thomas B. K. Watkins, Chris Bailey, Oriol Pich, Zhihui Zhang, Peter Van Loo, TRACERx Consortium, Robert Bentham, Thomas P. Jones, Maria Litovchenko, Kerstin Thol, Thomas B. K. Watkins, Chris Bailey, Oriol Pich, Mariam Jamal-Hanjani, Carlos Martínez-Ruiz, Peter Van Loo, James R. M. Black, Takahiro Karasaki, Abigail Bunkum, Sonya Hessey, Wing Kin Liu, Nicolai J. Birkbak, Alexander M. Frankell, Ariana Huebner, Clare Puttick, Crispin T. Hiley, David A. Moore, Dhruva Biswas, Emilia L. Lim, Kristiana Grigoriadis, Maise Al Bakir, Olivia Lucas, Roberto Vendramin, Sophia Ward, Sian Harries, Simone Zaccaria, Rija Zaidi, Lucrezia Patruno, Despoina Karagianni, Sergio A. Quezada, Supreet Kaur Bola, Martin D. Forster, Siow Ming Lee, Corentin Richard, Cristina Naceur-Lombardelli, Francisco Gimeno-Valiente, Krupa Thakkar, Monica Sivakumar, Nnennaya Kanu, Ieva Usaite, Sadegh Saghafinia, Selvaraju Veeriah, Sharon Vanloo, Antonia Toncheva, Paulina Prymas, Bushra Mussa, Michalina Magala, Elizabeth Keene, Michelle M. Leung, Gareth A. Wilson, Rachel Rosenthal, Andrew Rowan, Claudia Lee, Emma Colliver, Katey S. S. Enfield, Mihaela Angelova, Cian Murphy, Maria Zagorulya, Teresa Marafioti, Elaine Borg, Mary Falzon, Reena Khiroya, Yien Ning Sophia Wong, Emilie Martinoni Hoogenboom, Fleur Monk, James W. Holding, Junaid Choudhary, Kunal Bhakhri, Pat Gorman, Robert C. M. Stephens, Maria Chiara Pisciella, Steve Bandula, Jerome Nicod, Angela Dwornik, Angeliki Karamani, Benny Chain, David R. Pearce, Georgia Stavrou, Gerasimos-Theodoros Mastrokalos, Helen L. Lowe, James L. Reading, John A. Hartley, Kayalvizhi Selvaraju, Leah Ensell, Mansi Shah, Piotr Pawlik, Samuel Gamble, Seng Kuong Anakin Ung, Victoria Spanswick, Yin Wu, Jason F. Lester, Sean Dulloo, Dean A. Fennell, Amrita Bajaj, Apostolos Nakas, Azmina Sodha-Ramdeen, Mohamad Tufail, Molly Scotland, Rebecca Boyles, Sridhar Rathinam, Claire Wilson, Gurdeep Matharu, Jacqui A. Shaw, Ekaterini Boleti, Heather Cheyne, Mohammed Khalil, Shirley Richardson, Tracey Cruickshank, Gillian Price, Keith M. Kerr, Sarah Benafif, Dionysis Papadatos-Pastos, James Wilson, Tanya Ahmad, Jack French, Kayleigh Gilbert, Babu Naidu, Akshay J. Patel, Gary Middleton, Aya Osman, Mandeesh Sangha, Gerald Langman, Helen Shackleford, Madava Djearaman, Angela Leek, Jack Davies Hodgkinson, Nicola Totton, Philip Crosbie, Eustace Fontaine, Felice Granato, Juliette Novasio, Kendadai Rammohan, Leena Joseph, Paul Bishop, Vijay Joshi, Sara Waplington, Adam Atkin, Katherine D. Brown, Mathew Carter, Anshuman Chaturvedi, Pedro Oliveira, Colin R. Lindsay, Fiona H. Blackhall, Yvonne Summers, Matthew G. Krebs, Antonio Paiva-Correia, Jonathan Tugwood, Caroline Dive, Hugo J. W. L. Aerts, Roland F. Schwarz, Tom L. Kaufmann, Zoltan Szallasi, Miklos Diossy, Roberto Salgado, Jonas Demeulemeester, Carla Castignani, Stephan Beck, George Kassiotis, Imran Noorani, Clare E. Weeden, Eva Grönroos, Jacki Goldman, Mickael Escudero, Philip Hobson, Stefan Boeing, Tamara Denner, Vittorio Barbè, Wei-Ting Lu, William Hill, Yutaka Naito, Erik Sahai, Zoe Ramsden, Emma Nye, Richard Kevin Stone, Jayant K. Rane, Jeanette Kittel, Kerstin Haase, Kexin Koh, Rachel Scott, Karl S. Peggs, Catarina Veiga, Gary Royle, Charles-Antoine Collins-Fekete, Francesco Fraioli, Paul Ashford, Arjun Nair, Alexander James Procter, Asia Ahmed, Magali N. Taylor, David Lawrence, Davide Patrini, Neal Navani, Ricky M. Thakrar, Sam M. Janes, Zoltan Kaplar, Allan Hackshaw, Camilla Pilotti, Rachel Leslie, Anne-Marie Hacker, Sean Smith, Aoife Walker, Anca Grapa, Hanyun Zhang, Khalid AbdulJabbar, Xiaoxi Pan, Yinyin Yuan, David Chuter, Mairead MacKenzie, Serena Chee, Patricia Georg, Aiman Alzetani, Judith Cave, Eric Lim, Andrew G. Nicholson, Paulo De Sousa, Simon Jordan, Alexandra Rice, Hilgardt Raubenheimer, Harshil Bhayani, Lyn Ambrose, Anand Devaraj, Hema Chavan, Sofina Begum, Silviu I. Buderi, Daniel Kaniu, Mpho Malima, Sarah Booth, Nadia Fernandes, Pratibha Shah, Chiara Proli, Madeleine Hewish, Sarah Danson, Michael J. Shackcloth, Lily Robinson, Peter Russell, Kevin G. Blyth, Andrew Kidd, Craig Dick, John Le Quesne, Alan Kirk, Mo Asif, Rocco Bilancia, Nikos Kostoulas, Jennifer Whiteley, Mathew Thomas, Charles Swanton, Nicholas McGranahan, Genomics England Consortium, J. C. Ambrose, P. Arumugam, E. L. Baple, M. Bleda, F. Boardman-Pretty, J. M. Boissiere, C. R. Boustred, H. Brittain, M. J. Caulfield, G. C. Chan, C. E. H. Craig, L. C. Daugherty, A. de Burca, A. Devereau, G. Elgar, R. E. Foulger, T. Fowler, P. Furió-Tarí, J. M. Hackett, D. Halai, A. Hamblin, S. Henderson, J. E. Holman, T. J. P. Hubbard, K. Ibáñez, R. Jackson, L. J. Jones, D. Kasperaviciute, M. Kayikci, L. Lahnstein, L. Lawson, S. E. A. Leigh, I. U. S. Leong, F. J. Lopez, F. Maleady-Crowe, J. Mason, E. M. McDonagh, L. Moutsianas, M. Mueller, N. Murugaesu, A. C. Need, C. A. Odhams, C. Patch, D. Perez-Gil, D. Polychronopoulos, J. Pullinger, T. Rahim, A. Rendon, P. Riesgo-Ferreiro, T. Rogers, M. Ryten, K. Savage, K. Sawant, R. H. Scott, A. Siddiq, A. Sieghart, D. Smedley, K. R. Smith, A. Sosinsky, W. Spooner, H. E. Stevens, A. Stuckey, R. Sultana, E. R. A. Thomas, S. R. Thompson, C. Tregidgo, A. Tucci, E. Walsh, S. A. Watters, M. J. Welland, E. Williams, K. Witkowska, S. M. Wood, M. Zarowiecki, Charles Swanton, Nicholas McGranahan

**Affiliations:** 1 Cancer Genome Evolution Research Group, Cancer Research UK Lung Cancer Centre of Excellence, University College London Cancer Institute, London, UK; 2 Cancer Research UK Lung Cancer Centre of Excellence, University College London Cancer Institute, London, UK; 3 Cancer Evolution and Genome Instability Laboratory, The Francis Crick Institute, London, UK; 4 Department of Pathology, Stanford University School of Medicine, Stanford, CA USA; 5 Department of Genetics, The University of Texas MD Anderson Cancer Center, Houston, TX USA; 6 Department of Genomic Medicine, The University of Texas MD Anderson Cancer Center, Houston, TX USA; 7 Department of Medical Oncology, University College London Hospitals, London, UK; 8 Cancer Metastasis Laboratory, University College London Cancer Institute, London, UK; 9 Department of Thoracic Surgery, Respiratory Center, Toranomon Hospital, Tokyo, Japan; 10 Computational Cancer Genomics Research Group, University College London Cancer Institute, London, UK; 11 Department of Molecular Medicine, Aarhus University Hospital, Aarhus, Denmark; 12 Department of Clinical Medicine, Aarhus University, Aarhus, Denmark; 13 Bioinformatics Research Centre, Aarhus University, Aarhus, Denmark; 14 Department of Cellular Pathology, University College London Hospitals, London, UK; 15 Bill Lyons Informatics Centre, University College London Cancer Institute, London, UK; 16 University College London Hospitals, London, UK; 17 Tumour Immunogenomics and Immunosurveillance Laboratory, University College London Cancer Institute, London, UK; 18 Genomics Science Technology Platform, The Francis Crick Institute, London, UK; 19 Immune Regulation and Tumour Immunotherapy Group, Cancer Immunology Unit, Research Department of Haematology, University College London Cancer Institute, London, UK; 20 National Cancer Centre, Singapore City, Singapore; 21 University College London Cancer Institute, London, UK; 22 Singleton Hospital, Swansea Bay University Health Board, Swansea, UK; 23 University of Leicester, Leicester, UK; 24 University Hospitals of Leicester NHS Trust, Leicester, UK; 25 Leicester Medical School, University of Leicester, Leicester, UK; 26 Cancer Research Centre, University of Leicester, Leicester, UK; 27 Royal Free London NHS Foundation Trust, London, UK; 28 Aberdeen Royal Infirmary NHS Grampian, Aberdeen, UK; 29 Department of Medical Oncology, Aberdeen Royal Infirmary NHS Grampian, Aberdeen, UK; 30 University of Aberdeen, Aberdeen, UK; 31 Department of Pathology, Aberdeen Royal Infirmary NHS Grampian, Aberdeen, UK; 32 The Whittington Hospital NHS Trust, London, UK; 33 Birmingham Acute Care Research Group, Institute of Inflammation and Ageing, University of Birmingham, Birmingham, UK; 34 Guy’s and St Thomas’ NHS Foundation Trust, London, UK; 35 University Hospital Birmingham NHS Foundation Trust, Birmingham, UK; 36 Institute of Immunology and Immunotherapy, University of Birmingham, Birmingham, UK; 37 Manchester Cancer Research Centre Biobank, Manchester, UK; 38 Wythenshawe Hospital, Manchester University NHS Foundation Trust, Wythenshawe, UK; 39 Division of Infection, Immunity and Respiratory Medicine, University of Manchester, Manchester, UK; 40 Cancer Research UK Lung Cancer Centre of Excellence, University of Manchester, Manchester, UK; 41 The Christie NHS Foundation Trust, Manchester, UK; 42 Division of Cancer Sciences, The University of Manchester and The Christie NHS Foundation Trust, Manchester, UK; 43 Manchester University NHS Foundation Trust, Manchester, UK; 44 CRUK Manchester Institute Cancer Biomarker Centre, University of Manchester, Manchester, UK; 45 Artificial Intelligence in Medicine (AIM) Program, Mass General Brigham, Harvard Medical School, Boston, MA USA; 46 Department of Radiation Oncology, Brigham and Women’s Hospital, Dana–Farber Cancer Institute, Harvard Medical School, Boston, MA USA; 47 Radiology and Nuclear Medicine, CARIM & GROW, Maastricht University, Maastricht, The Netherlands; 48 Institute for Computational Cancer Biology, Center for Integrated Oncology (CIO), Cancer Research Center Cologne Essen (CCCE), Faculty of Medicine and University Hospital Cologne, University of Cologne, Cologne, Germany; 49 Berlin Institute for the Foundations of Learning and Data (BIFOLD), Berlin, Germany; 50 Berlin Institute for Medical Systems Biology, Max Delbrück Center for Molecular Medicine in the Helmholtz Association (MDC), Berlin, Germany; 51 Danish Cancer Institute, Copenhagen, Denmark; 52 Computational Health Informatics Program, Boston Children’s Hospital, Boston, MA USA; 53 Department of Bioinformatics, Semmelweis University, Budapest, Hungary; 54 Department of Physics of Complex Systems, ELTE Eötvös Loránd University, Budapest, Hungary; 55 Department of Pathology, ZAS Hospitals, Antwerp, Belgium; 56 Division of Research, Peter MacCallum Cancer Centre, Melbourne, Victoria Australia; 57 Integrative Cancer Genomics Laboratory, VIB Center for Cancer Biology, Leuven, Belgium; 58 VIB Center for AI & Computational Biology, Leuven, Belgium; 59 Department of Oncology, KU Leuven, Leuven, Belgium; 60 Cancer Genomics Laboratory, The Francis Crick Institute, London, UK; 61 Medical Genomics, University College London Cancer Institute, London, UK; 62 The Francis Crick Institute, London, UK; 63 Department of Infectious Disease, Faculty of Medicine, Imperial College London, London, UK; 64 Department of Neurosurgery, National Hospital for Neurology and Neurosurgery, London, UK; 65 Institute of Neurology, University College London, London, UK; 66 Experimental Histopathology, The Francis Crick Institute, London, UK; 67 Department of Haematology, University College London Hospitals, London, UK; 68 Cancer Immunology Unit, Research Department of Haematology, University College London Cancer Institute, London, UK; 69 Centre for Medical Image Computing, Department of Medical Physics and Biomedical Engineering, London, UK; 70 Department of Medical Physics and Bioengineering, University College London Cancer Institute, London, UK; 71 Institute of Nuclear Medicine, Division of Medicine, University College London, London, UK; 72 Institute of Structural and Molecular Biology, University College London, London, UK; 73 Department of Radiology, University College London Hospitals, London, UK; 74 UCL Respiratory, Department of Medicine, University College London, London, UK; 75 Department of Thoracic Surgery, University College London Hospital NHS Trust, London, UK; 76 Department of Thoracic Medicine, University College London Hospitals, London, UK; 77 Lungs for Living Research Centre, UCL Respiratory, Department of Medicine, University College London, London, UK; 78 Integrated Radiology Department, North-Buda St John’s Central Hospital, Budapest, Hungary; 79 Institute of Nuclear Medicine, University College London Hospitals, London, UK; 80 Cancer Research UK & UCL Cancer Trials Centre, London, UK; 81 The Institute of Cancer Research, London, UK; 82 Garvan Institute of Medical Research, Sydney, New South Wales Australia; 83 Case45, London, UK; 84 The University of Texas MD Anderson Cancer Center, Houston, TX USA; 85 Independent Cancer Patient’s Voice, London, UK; 86 University Hospital Southampton NHS Foundation Trust, Southampton, UK; 87 The NIHR Southampton Biomedical Research Centre, University Hospital Southampton NHS Foundation Trust, Southampton, UK; 88 Department of Oncology, University Hospital Southampton NHS Foundation Trust, Southampton, UK; 89 Academic Division of Thoracic Surgery, Imperial College London, London, UK; 90 Royal Brompton and Harefield Hospitals, Part of Guy’s and St Thomas’ NHS Foundation Trust, London, UK; 91 National Heart and Lung Institute, Imperial College, London, UK; 92 Royal Surrey Hospital, Royal Surrey Hospitals NHS Foundation Trust, Guildford, UK; 93 University of Surrey, Guildford, UK; 94 University of Sheffield, Sheffield, UK; 95 Sheffield Teaching Hospitals NHS Foundation Trust, Sheffield, UK; 96 Liverpool Heart and Chest Hospital, Liverpool, UK; 97 Princess Alexandra Hospital, The Princess Alexandra Hospital NHS Trust, Harlow, UK; 98 School of Cancer Sciences, University of Glasgow, Glasgow, UK; 99 Beatson Institute for Cancer Research, University of Glasgow, Glasgow, UK; 100 Queen Elizabeth University Hospital, Glasgow, UK; 101 Institute of Infection, Immunity & Inflammation, University of Glasgow, Glasgow, UK; 102 NHS Greater Glasgow and Clyde, Glasgow, UK; 103 Cancer Research UK Scotland Institute, Glasgow, UK; 104 Institute of Cancer Sciences, University of Glasgow, Glasgow, UK; 105 NHS Greater Glasgow and Clyde Pathology Department, Queen Elizabeth University Hospital, Glasgow, UK; 106 Golden Jubilee National Hospital, Clydebank, UK; 107 Genomics England, London, UK; 108 William Harvey Research Institute, Queen Mary University of London, London, UK

**Keywords:** Tumour immunology, DNA sequencing, Software, Genetics research

## Abstract

Recognition and elimination of pathogens and cancer cells depend on the adaptive immune system. Thus, accurate quantification of immune subsets is vital for precision medicine. We present immune lymphocyte estimation from nucleotide sequencing (ImmuneLENS), which estimates T cell and B cell fractions, class switching and clonotype diversity from whole-genome sequencing data at depths as low as 5× coverage. By applying ImmuneLENS to the 100,000 Genomes Project, we identify genes enriched with somatic mutations in T cell-rich tumors, significant sex-based differences in circulating T cell fraction and demonstrated that the circulating T cell fraction in patients with cancer is significantly lower than in healthy individuals. Low circulating B cell fraction was linked to increased cancer incidence. Finally, circulating T cell abundance was more prognostic of 5-year cancer survival than infiltrating T cells.

## Abstract (web summary)

Immune lymphocyte estimation from nucleotide sequencing (ImmuneLENS) infers B cell and T cell fractions from whole-genome sequencing data. Applied to the 100,000 Genomes Project datasets, circulating T cell fraction provides sex-dependent and prognostic insights in patients.

## Main

Measuring the quantity, quality and location of immune cells is vital to understanding their role and function. In cancer research, studies have focused on tumor-infiltrating lymphocytes and their roles in cancer evolution and immune evasion^1–5^.

High tumor T cell infiltration is prognostic in cancer^6,7^ and influences immunotherapy response^8^. However, B cell infiltration has been linked to both cancer promotion and inhibition^9^. Classifying tumor-infiltrating B cells into different lineages might elucidate their role in cancer, but such analysis without direct assays of B cell markers with flow cytometry, targeted B cell receptor repertoire sequencing or single-cell RNA sequencing (RNA-seq) is challenging.

Although circulating immune cell counts from routine blood tests have been associated with response to therapy and prognosis in cancer^10,11^, a systematic exploration of their relative importance compared to tumor-infiltrating immune cells and their clinical correlates is lacking.

Here we present immune lymphocyte estimation from nucleotide sequencing (ImmuneLENS)—a method to quantify immune content from whole-genome sequencing (WGS) data and available at https://github.com/McGranahanLab/ImmuneLENS. In addition to T cell content, our method predicts B cell fraction and class switching from the *IGH* locus and provides estimates for both T cell receptor (TCR) and B cell receptor (BCR) diversity.

## Results

### Inferring T cell and B cell fractions from WGS data

WGS enables in-depth characterization of somatic mutations, structural variants and copy number alterations^12^. However, whether WGS can simultaneously be used for the estimation of lymphocyte infiltration has not been extensively evaluated.

**Fig. 1:**
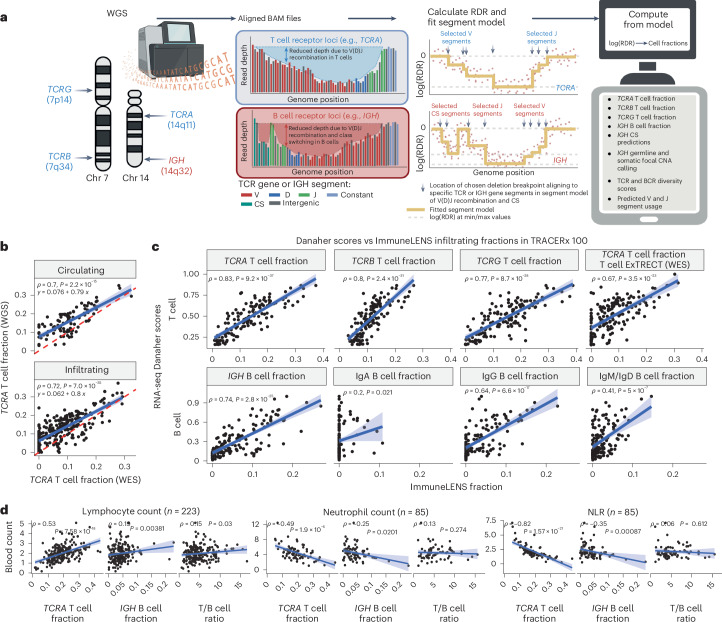
Overview of ImmuneLENS and validation. **a**, Overview of the ImmuneLENS method. The figure is created with BioRender.com. **b**, Scatter plot of *TCRA* T cell fractions calculated from TRACERx WGS versus WES data. The red dotted line represents *y* = *x*; the blue line shows the line of best fit with a light blue-shaded 95% confidence interval (CI). **c**, Scatter plots comparing ImmuneLENS fractions from TRACERx100 WGS and *TCRA* T cell fractions from T cell ExTRECT (TRACERx100 WES data) against T cell- and B cell-related Danaher scores from matched RNA-seq samples. The blue line represents the line of best fit with a light blue-shaded 95% CI. **d**, Correlation of *TCRA* T cell fraction, *IGH* B cell fraction and T/B cell ratio with date-matched blood count data (lymphocyte count, neutrophil count and NLR values) within the 100KGP cohort. The blue line represents the line of best fit with a light blue-shaded 95% CI. *P* values for Spearman’s *ρ* were derived from a two-tailed *t* distribution using the correlation coefficient and sample size. CNA, copy number alteration; CS, class switching; RDR, read depth ratio.

**Extended Data Fig. 1:**
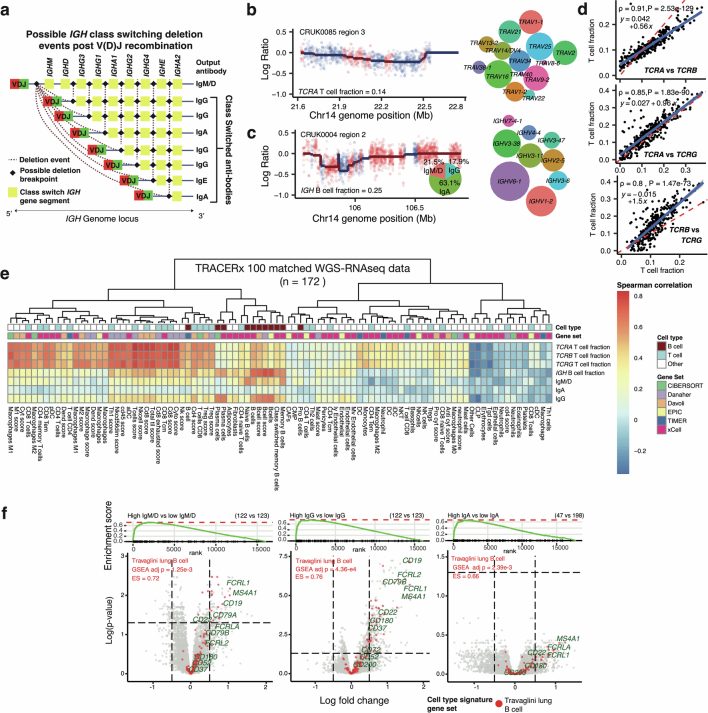
Validation and description of ImmuneLENS. **a**, Diagram illustrating possible class-switching deletion events following VDJ recombination at the *IGH* locus, resulting in B cells producing different antibodies. **b**, Example ImmuneLENS output showing the *TCRA* locus for TRACERx sample CRUK0085 region 3. The log read depth ratio plot corresponds to a predicted T cell fraction of 0.14. Alternating colors represent changes in the 14 TRAV segments selected by the model, which are also depicted in the bubble plot (right). **c**, Example ImmuneLENS output showing the *IGH* locus for TRACERx sample CRUK0004 region 2, with a predicted B cell fraction of 0.25. IGHV segment usage and class-switching percentages are also displayed (right). **d**, Scatter plots showing the correlation between T cell fraction values calculated by ImmuneLENS from the *TCRA*, *TCRB* or *TCRG* loci. The blue line represents the line of best fit, and the gray region indicates the 95% confidence interval. **e**, Heatmap of ImmuneLENS’ fractions compared to RNA-seq signatures for different cell types. **f**, Differential gene expression analysis (bottom) of TRACERx RNA-seq samples split into high and low groups based on median predicted non-class-switched IgM/IgD B cell fractions and class-switched IgA and IgG B cell fractions. Analysis and significance were assessed using limma–voom (Methods), accounting for multiple hypothesis testing. Red points represent genes within the Travaglini lung B cell gene signature^59^. P values were derived from a GSEA analysis (top) of all cell type signature genesets defined by MSigDB, with P-value estimation based on an adaptive multi-level split Monte Carlo scheme. The P values for Spearman’s ρ in **d** were derived from a two-tailed t-distribution using the correlation coefficient and sample size.

To this end, we created ImmuneLENS. This tool significantly enhances and extends our previous method, T cell ExTRECT^6^ (see Methods and Fig. 1a for an overview), which was designed for whole-exome sequencing (WES). We made three improvements to the method. First, we introduced a segment-based model, enabling precise breakpoint fitting based on the locations of individual V and J segments. Second, harnessing the predicted V and J segment usage, the model estimates both lymphocyte cell fractions and clonotype diversity. Finally, we introduced an estimate of B cell fraction and immunoglobulin (Ig) class switching from the *IGH* locus, enabling distinction between non-class-switched B cells (IgM/IgD) and class-switched B cells that produce IgA, IgG or IgE antibodies (Extended Data Fig. 1a and Supplementary Fig. 1). The B cell fraction model also incorporates an *IGH* locus germline copy number variant caller (Supplementary Fig. 2). Example output of ImmuneLENS is illustrated in Extended Data Fig. 1b,c.

### WGS enables accurate measurement of T cell and B cell fraction

We first evaluated the accuracy of T cell fractions predicted by ImmuneLENS on TRACERx100 (ref. ^13^) lung cancer samples that had both matched WES and WGS (*n* = 322) or orthogonal RNA-seq data (*n* = 126).

WES and WGS *TCRA* T cell fractions showed positive correlations in both blood (*ρ* = 0.70, *P* = 2.2 × 10^−15^) and tumor samples (*ρ* = 0.72, *P* = 7.0 × 10^−35^; Fig. 1b). Notably, fewer samples exhibited no T cell infiltrate in WGS than WES (54 WES samples had <10^−4^ T cell fraction compared to only two of the WGS samples), likely reflecting ImmuneLENS’ increased sensitivity (Methods; Supplementary Fig. 3).

αβ T cells have recombined TCRα and TCRβ (encoded by *TCRB*) chains, whereas γδ Τ cells have TCRγ (encoded by *TCRG*) and TCRδ chains (encoded by *TCRD*). *TCRB* and *TCRG* can provide T cell fraction estimates independent of *TCRA*. The T cell fraction estimates of these distinct T cell classes were all positively correlated with each other, indicating that each independently measures T cell content (*ρ* > 0.8, *P* < 10^−^^70^; Extended Data Fig. 1d). There are two important caveats to this result. First, *TCRB* T cell fraction was systematically smaller than *TCRA* (line of best fit: *y* = 0.56× + 0.042), likely due to allelic exclusion^14^. Second, *TCRG*, which is typically expressed only in γδ T cells (1–5% of CD3^+^ T cells^15^), strongly correlated with *TCRA*, suggesting αβ T cells commonly retain rearranged *TCRG* loci. Previous reports have shown that αβ T cells frequently rearrange their *TCRG* locus before committing to the αβ lineage^16^. Thus, *TCRG* appears to measure total T cell fraction rather than solely γδ T cells.

We further validated ImmuneLENS using TRACERx100 RNA-seq data (*n* = 126). *TCRA*, *TCRB* and *TCRG* T cell fractions strongly correlated with the Danaher T cell signature^17^, previously shown to reflect T cell content^18^ (*TCRA*: *ρ* = 0.83, *P* = 9.2 × 10^−37^; *TCRB*: *ρ* = 0.8, *P* = 2.4 × 10^−31^; *TCRG*: *ρ* = 0.77, *P* = 8.7 × 10^−28^; Fig. 1c). Consistent correlations were also observed with RNA-seq signatures from TIMER^19^, CIBERSORT^20^, xCell^20^ and scores from ref. ^21^ (Extended Data Fig. 1e). Likewise, *IGH* B cell fraction strongly correlated with Danaher RNA-seq-based B cell score (Fig. 1c; *ρ* = 0.74, *P* = 2.8 × 10^−25^). However, we observed that correlation strength varied by class—IgG (*ρ* = 0.64, *P* = 6.6 × 10^−17^), IgM/IgD (*ρ* = 0.41, *P* = 5 × 10^−7^) and IgA (*ρ* = 0.2, *P* = 0.021). Thus, conceivably IgA B cells may be underrepresented in RNA-seq data or overestimated in DNA (Supplementary Figs. 4 and 5). These results aligned with other RNA-seq-based B cell signatures (Extended Data Fig. 1e). Samples with high WGS-inferred B cell fractions (>median) showed significant enrichment of B cell gene expression for all subsets, even IgA (IgG: adjusted *P* = 4.4 × 10^−4^; IgM/IgD: adjusted *P* = 1.3 × 10^−3^; IgA: adjusted *P* = 2.4 × 10^−3^; Extended Data Fig. 1f).

**Extended Data Fig. 2:**
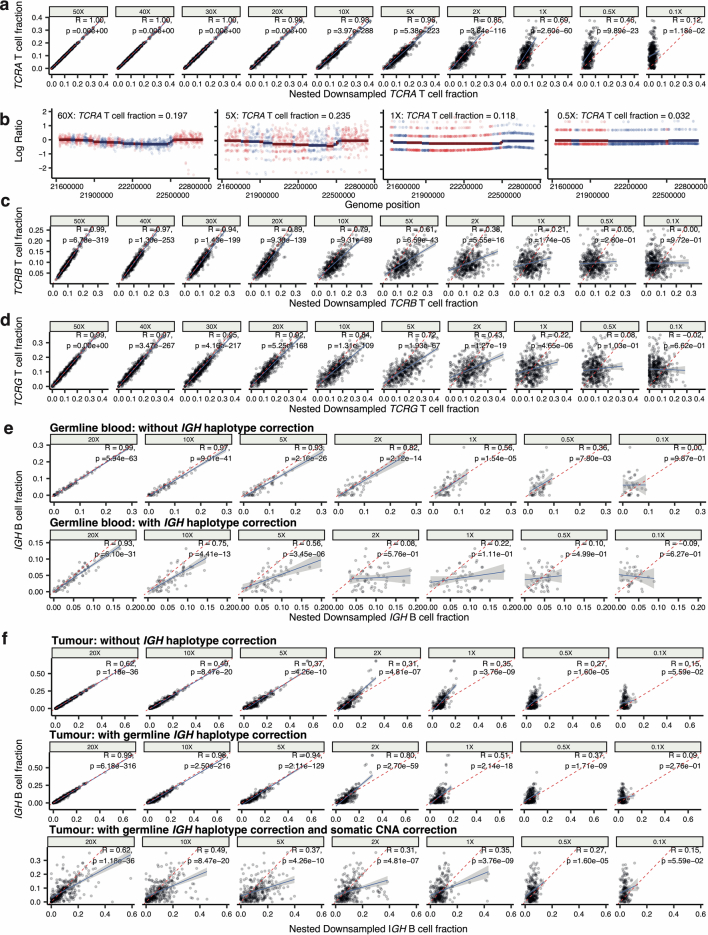
Nested downsampling of TRACERx data. **a**, Correlation of T cell fraction at 60× coverage with samples downsampled to different coverage levels using nested downsampling. **b**, Example ImmuneLENS model outputs of the same sample at different downsampled coverage depths. **c**,**d**. Correlation of *TCRB* and *TCRG* T cell fractions at 60× coverage with samples downsampled to different coverage levels using nested downsampling. **e**, Correlation of circulating B cell fraction at 30× coverage with samples downsampled to different coverage levels using nested downsampling. Top: B cell fraction calculated without *IGH* haplotype correction. Bottom: B cell fraction calculated with *IGH* haplotype correction **f**, Correlation of infiltrating B cell fraction at 30× coverage with samples downsampled to different coverage levels using nested downsampling. Top: B cell fraction calculated without *IGH* haplotype correction. Middle: B cell fraction calculated with germline *IGH* haplotype correction. Bottom: B cell fraction calculated with both germline and somatic *IGH* haplotype correction. Throughout, blue lines represent the line of best fit, and gray regions indicate the 95% confidence interval. The P values for Pearson’s R were derived from a two-tailed t-distribution using the correlation coefficient and sample size.

ImmuneLENS provided accurate T cell measurements at depths as low as 5× for *TCRA* (*R* = 0.96, *P* = 5.4 × 10^−223^), *TCRB* (*R* = 0.61, *P* = 6.59 × 10^−43^) and *TCRG* (*R* = 0.72, *P* = 1.93 × 10^−67^) as evidenced using downsampled data (Extended Data Fig. 2a–d). For B cell quantification, >10× was required for accurate germline copy number inference (Extended Data Fig. 2e). Similarly, correction for possible tumor copy number alterations was only accurate at depths >20× (Extended Data Fig. 2f). Consistent results were observed for matched high and low-coverage WGS data (Supplementary Fig. 6).

**Extended Data Fig. 3:**
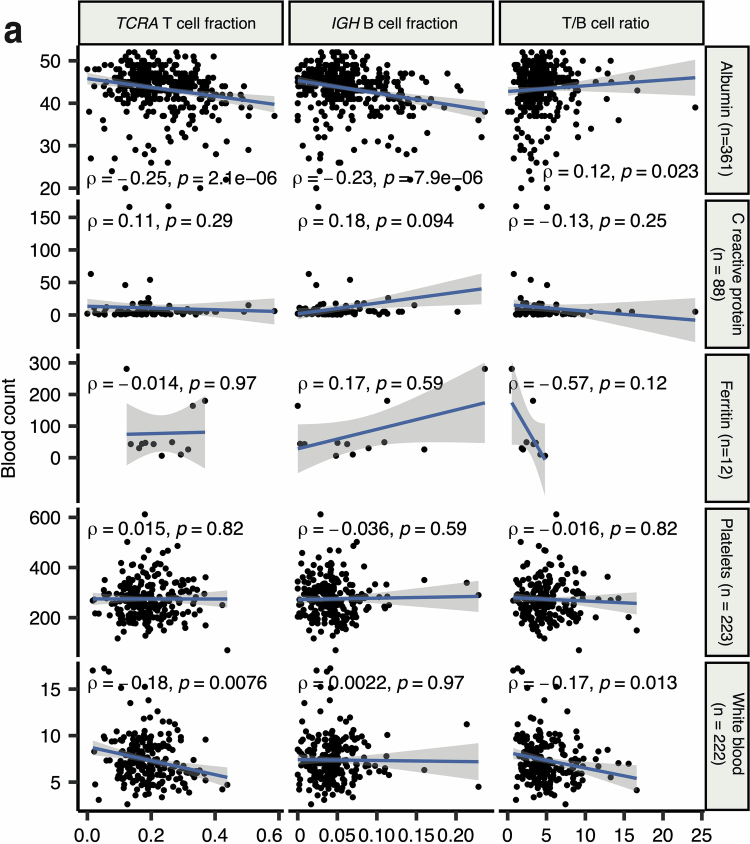
Association of ImmuneLENS scores with blood count data. **a**. ImmuneLENS fractions versus date-matched blood count data for albumin, C-reactive protein, ferritin, platelets and white blood count. Blue lines represent the line of best fit with gray regions representing 95% confidence interval. P values for Spearman’s ρ were derived from a two-tailed t-distribution using the correlation coefficient and sample size.

To further validate ImmuneLENS, we applied the tool to blood-derived WGS samples with date-matched blood count data from 441 participants of the 100,000 Genomes Project (100KGP; Methods). Circulating T cell fraction correlated positively with lymphocyte count (*ρ* = 0.53, *P* = 7.6 × 10^−18^) and negatively with both neutrophil count (*ρ* = −0.49, *P* = 1.9 × 10^−6^) and neutrophil-to-lymphocyte ratio (NLR) (*ρ* = −0.82, *P* = 1.6 × 10^−21^; Fig. 1d). These data suggest that *TCRA* T cell fraction serves as an NLR proxy. A weak negative correlation with albumin concentration was observed (Extended Data Fig. 3a; *ρ* = −0.25, *P* = 2.1 × 10^−6^). No significant associations were found with C-reactive protein or ferritin (Extended Data Fig. 3a), although weak negative correlations existed between white blood cell count and both T cell fraction (*ρ* = −0.18, *P* = 0.0076) and T/B cell ratio (*ρ* = −0.17, *P* = 0.013). Similar trends were observed for *IGH* B cell fraction (Fig. 1d). Notably, the T/B cell ratio correlated significantly with lymphocyte count (*ρ* = 0.15, *P* = 0.03) but not with neutrophil count (*ρ* = 0.13, *P* = 0.27) or NLR (*ρ* = 0.06, *P* = 0.612; Fig. 1d). Thus, the T/B cell ratio provides a measure of lymphocyte count, which is independent of neutrophil levels.

### Investigating T cell receptor diversity from WGS data

**Extended Data Fig. 4:**
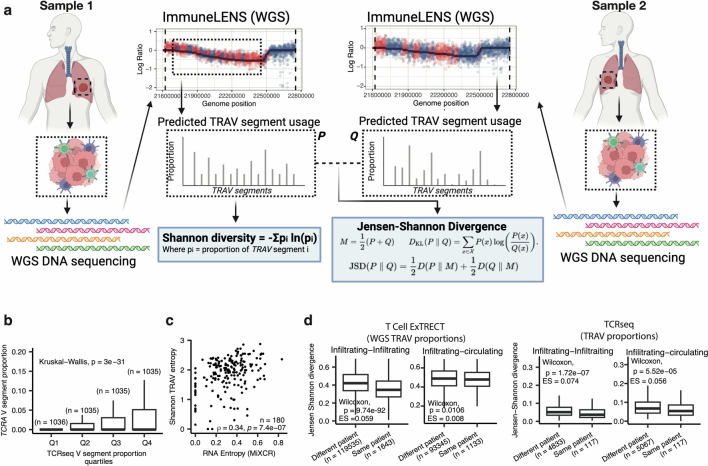
Validation of TCR diversity metrics. **a**, Cartoon overview of the use of ImmuneLENS to calculate Shannon diversity values from TRAV segment usage as well as divergence metrics between two samples using the Jensen–Shannon divergence (JSD) from TRAV segment usage. The figure is created with BioRender.com. **b**, Proportion of TRAV segment usage in samples as measured from either TRACERx TCR-seq data (separated into 4 quartiles) or predicted by ImmuneLENS. **c**, Correlation of Shannon entropy measurements as measured by either TRAV segments predicted by ImmuneLENS or from MiXCR (RNA-seq). **d**, Jensen–Shannon divergence of samples either from different or same TRACERx patients, for tumor–tumor or tumor–blood sample comparisons measured by ImmuneLENS or TCR-seq from TRAV segment proportions, with significance assessed using a two-sided Wilcoxon rank-sum test. Boxplots in **b** and **d** show the median, lower and upper quartile and with whiskers extending to 1.5× the interquartile range above and below the interquartile range. P values for Spearman’s ρ were derived from a two-tailed t-distribution using the correlation coefficient and sample size.

The ability of ImmuneLENS to fit individual V and J segments allows TCR and BCR diversity analysis from WGS data (Extended Data Fig. 4a).

We assessed TCR diversity accuracy from WGS data using three methods. First, we compared ImmuneLENS output with matched TCR-sequencing (TCR-seq) data in TRACERx. ImmuneLENS’ T cell receptor alpha variable (TRAV) segment proportions were significantly different between quartiles representing different levels of actual segment usage inferred from TCR-seq (Kruskal–Wallis, *P* = 3.3 × 10^−31^; Extended Data Fig. 4b). ImmuneLENS likely underestimates TCR diversity, illustrated by low TRAV segment usage predictions in Extended Data Fig. 4b. Second, we compared Shannon diversity scores from WGS-derived V segment usage (ImmuneLENS) with the calculated TCR repertoire diversity from RNA-seq data (using MiXCR^22^). A significant correlation was observed between DNA- and RNA-derived TCR diversity scores (Extended Data Fig. 4c; *ρ* = 0.34, *P* = 7.4 × 10^−7^). Third, using the Jensen–Shannon divergence, we found significantly more similar repertoires in samples from the same patient than from different patients, for both infiltrating–infiltrating and infiltrating–circulating comparisons (Wilcoxon rank-sum *P* = 9.74 × 10^−92^ and *P* = 0.011, respectively; Extended Data Fig. 4d).

These results therefore demonstrate that predicted TRAV segments enable the assessment of TCR diversity within samples and enable TCR repertoire comparisons across samples.

### The immune landscape in the 100KGP cohort

**Fig. 2:**
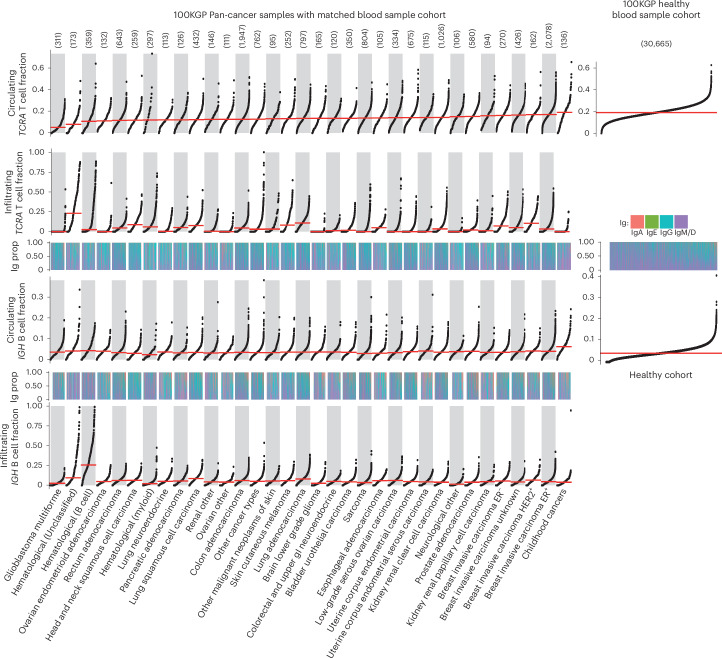
ImmuneLENS applied to 100KGP. The number of tumor samples per cancer histology is given above the plot. The panels represent snake plots for circulating and infiltrating *TCRA* T cell fractions and *IGH* B cell fractions, with each point representing a single blood or tumor sample. Above each *IGH* B cell fraction snake plot is a track, shown as a heatmap, displaying the proportion of different Ig B cells for each sample. Histology groups are arranged in ascending order based on the median circulating *TCRA* T cell fraction, and within each group, samples are sorted from lowest to highest value in each snake plot. Right, snake plots for the circulating T cell and B cell fractions within the 100KGP healthy cohort. No significant differences were identified (using ANOVA) in the proportions of B cell Ig status among cancer histology groups in either circulating or infiltrating samples. Horizontal red lines represent the median value per histology group. GI, gastrointestinal.

Having validated the accuracy of ImmuneLENS, we next applied the tool to 90,232 WGS samples from the 100KGP cohort^23^ (see Fig. 2a and Supplementary Data for a full clinical overview). This included 14,501 cancer samples across 33 distinct histologies (>100 participants each), with 13,870 having matched blood samples. In total, 631 cancer samples, including 538 with hematological cancers, lacked matched blood samples. Additionally, blood samples from 30,665 healthy individuals sequenced as relatives within the 100KGP rare disease cohort were analyzed. The remaining 30,565 WGS germline samples from the 100KGP rare disease cohort were excluded from our main analysis because they originated from either the rare disease probands or non-blood samples. We analyzed the following two measures: infiltrating immune cell fraction (from tumor WGS samples) and circulating immune cell fraction (from the buffy coat of blood WGS samples).

**Extended Data Fig. 5:**
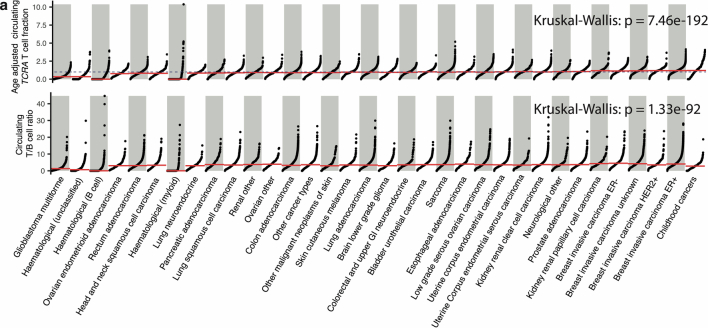
Pan-cancer overview of IGH and T/B cell ratio. **a**. Overview snake plot of age-adjusted *TCRA* T cell fraction and circulating T/B cell ratio, with horizontal red line representing the median value per histology and dashed black line at y = 1 for the age-adjusted circulating *TCRA* T cell fraction to show the median value expected for the age distribution of that cohort.

Significant differences in T cell fractions were observed between cancer types for both circulating and infiltrating T cells (Kruskal–Wallis, *P* = 5.3 × 10^−209^ and *P* = 3.7 × 10^−490^, respectively). Circulating T cell fractions were highest in patients with childhood cancer (median = 0.19) and lowest in patients with glioblastoma (median = 0.051). The low circulating T cell fraction in patients with glioblastoma may reflect steroid treatment, which increases circulating neutrophil levels^24^. Likewise, both infiltrating and circulating B cell fractions differed significantly across cancer types (Kruskal–Wallis, *P* = 3.8 × 10^−^^291^ and *P* = 3.3 × 10^−42^, respectively; Fig. 2). We found similar significant differences between cancer types for infiltrating T cells when adjusted by age and for the T/B cell ratio (Extended Data Fig. 5a). Additionally, consistent results in the Pan-Cancer Analysis of Whole Genomes (PCAWG)^12^ and The Cancer Genome Atlas (TCGA) WGS and WES datasets were identified (Supplementary Fig. 7).

**Extended Data Fig. 6:**
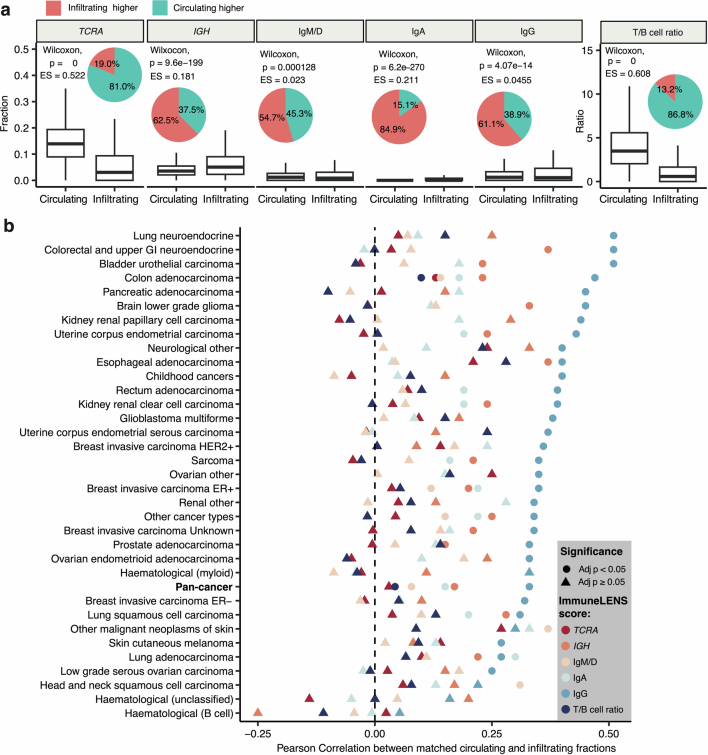
Comparison of circulating and infiltrating fractions. **a**, Boxplots showing different fractions calculated in ImmuneLENS in circulating blood and infiltrating tumor samples; pie charts show the percentage of cases when infiltrating fractions is higher or lower than circulating with significance assessed using a two-sided Wilcoxon rank-sum test. P = 0, represents P values less than the limit of double-precision floating numbers in R, 2.22 × 10^−^^308^ **b**, Pearson correlation of infiltrating and circulating fractions within the 100KGP cohort. Dashed black line represents the Pearson correlation = 0 and separates positive and negative correlations. Multiple hypothesis adjustments were performed using the Holm–Bonferroni method. Boxplots in **a** show the median, lower and upper quartile and with whiskers extending to 1.5× interquartile range above and below the interquartile range. P values for Pearson’s R values were derived from a two-tailed t-distribution using the correlation coefficient and sample size.

Tumor samples exhibited higher B cell content compared to blood samples (effect size = 0.181, *P* = 9.6 × 10^−199^; Extended Data Fig. 6a); conversely, T cell content was higher in blood samples than in tumors (effect size = 0.522, *P* < 2.22 × 10^−^^308^; Extended Data Fig. 6a). Circulating and infiltrating T cell fractions showed no clear correlation (*R* = 0.03, adjusted *P* = 0.068), except in colorectal adenocarcinoma (*R* = 0.13, adjusted *P* = 6.9 × 10^−7^; Extended Data Fig. 6b).

ImmuneLENS revealed differences in B cell class switching between infiltrating and circulating B cells across cancer types (Fig. 2a). Elevated tumor-infiltrating B cell levels compared to circulating were primarily due to an enrichment in class-switched IgA and IgG B cells (IgA: effect size = 0.21, *P* = 6.2 × 10^−270^; IgG: effect size = 0.046, *P* = 4.1 × 10^−14^; Extended Data Fig. 6a). IgM/IgD B cells showed a smaller but significant difference between circulating and infiltrating fractions (IgM/IgD: effect size = 0.023, *P* = 1.3 × 10^−^^4^; Extended Data Fig. 6a). This highlights key differences in the function and make-up of circulating and tumor-infiltrating B cells, underscoring the specialized roles of B cell subtypes. For instance, IgM antibodies in the circulatory system have an important role in activating the complement system, while in mucosal tissue, IgA antibodies are crucial for immune homeostasis. Consistent with these findings, we observed a significantly higher T/B cell ratio in blood compared to tumor samples (effect size = 0.61, *P* < 2.22 × 10^−308^; Extended Data Fig. 6a).

*IGH* B cell fractions, particularly IgG, correlated strongly between circulating and infiltrating in the majority of histologies (pan-cancer *IGH*: *R* = 0.17, adjusted *P* = 2.7 × 10^−^^86^; see full results in Supplementary Data and Extended Data Fig. 6b). Additionally, we found that TRAV segment usage differed significantly between circulating and infiltrating T cells and across cancer types, with *TRAV1–2* enriched in mucosal cancers. This may reflect mucosal-associated invariant T cells, a subset of T cells that recognize bacterial-produced metabolites that exclusively use the *TRAV1–2* segment^25^ (Supplementary Data and Supplementary Fig. 8).

### Determinants of circulating leukocyte fraction

Given the wide range of circulating immune fractions across both healthy participants and patients with cancer, we next sought to investigate the key determinants of leukocyte fraction.

**Fig. 3:**
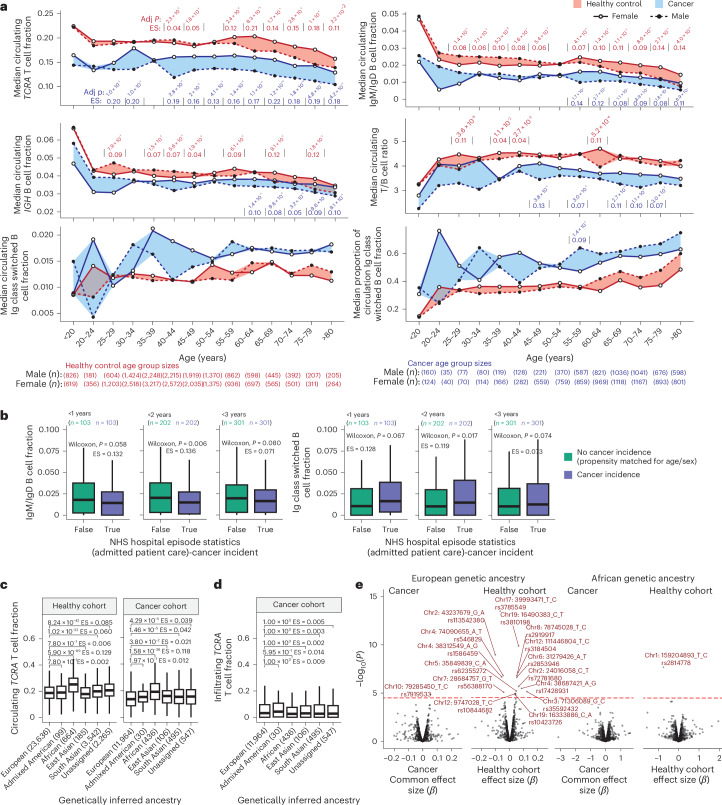
Disruption of circulating T cell fraction in patients with cancer. **a**, Ribbon plots of ImmuneLENS-related fractions in 5-year age brackets, split by the healthy control and cancer cohorts. The width of bands represents the extent of sexual dimorphism between male and female individuals, with significance assessed by two-sided Wilcoxon rank-sum tests within each age group and adjusted *P* values with effect size (ES) values shown. **b**, Boxplots of IgM/IgD and Ig class-switched B cell fractions from a subset of the healthy cohort with recorded cancer incidence post-WGS sequencing (from hospital episode statistics), compared to an age- and sex-matched propensity cohort of the same size. **c**, Boxplots of blood *TCRA* T cell fraction versus genetically inferred ancestry in the 100KGP healthy and cancer cohorts. **d**, Boxplots for tumor *TCRA* T cell fraction versus genetically inferred ancestry in the 100KGP cancer cohort. **e**, Volcano plots of known GWAS SNP associations with circulating *TCRA* T cell fraction. Multiple hypothesis adjustments were performed using the Benjamini–Hochberg method. Boxplots in **b**–**d** show the median and lower and upper quartiles, with whiskers extending to 1.5× interquartile range. Two-sided Wilcoxon rank-sum tests were used to assess the significance between groups in **b**–**d**. The *P* values in **e** are derived from PLINK software, which uses a linear regression model and performs a Wald test for each SNP. For the cancer cohort, this was done separately for each histology, and *P* values were combined using a meta-analysis with a common effects model.

Analysis of 100KGP participants in 5-year age brackets revealed declining T cell and B cell fractions with age in both healthy and cancer cohorts. For B cells, this effect was strongest for the IgM/IgD B cells, with the relative proportion of class-switched B cells increasing with age (Fig. 3a). Patients with cancer consistently exhibited lower circulating T cell and B cell fractions and higher class-switched B cell proportions compared to healthy individuals. Notably, on average a 40–45-year-old female patient with cancer has similar median circulating T cell fraction levels to a healthy >80-year-old female (0.161 versus 0.157). The decreased T/B cell ratio in the blood of patients with cancer suggests that this effect extends beyond a relative increase in neutrophils. Thus, in both healthy individuals and those with cancer, age is a key determinant of circulating immune fractions, and patients with cancer exhibit an inflated ‘immunological age’.

Sex differences in circulating immune fractions were evident in both cancer and healthy cohorts. Female patients with cancer showed significantly higher T cell fractions than male patients across most age groups (adjusted *P* < 0.001 for all age groups >40 years; Fig. 3a and Supplementary Data). In the healthy cohort, significant (adjusted *P* < 0.001) sex differences were primarily observed in older age groups (>55 years; Fig. 3a). Likewise, the T/B cell ratio difference between sexes was more pronounced in people with cancer, particularly in the 65–69 (adjusted *P* = 2.7 × 10^−5^, effect size = 0.11) and 70–74 age groups (adjusted *P* = 1.1 × 10^−^^4^, effect size = 0.10), compared to the healthy cohort (significant at adjusted *P* < 0.001 only in the 60–64 age group, adjusted *P* = 5.2 × 10^−^^4^, effect size = 0.11). This suggests neutrophil count may contribute to T cell fraction differences in the healthy cohort. For the IgM/IgD B cell fraction in the healthy group, a sex-based switch was observed from age 55, with male individuals higher initially and female individuals after age 55. In the cancer cohort, female patients exhibited higher B cell fractions in age groups >55. These trends were generally consistent across individual cancer types (Supplementary Fig. 9 and Supplementary Data).

To assess whether circulating immune cell levels could predict future cancer incidence, we identified 301 participants within the 100KGP healthy cohort who developed cancer within 3 years following germline blood sequencing. Compared to a set of controls propensity-matched for age and sex, those diagnosed with cancer within 2 but not 3 years showed significantly lower IgM/IgD (2 years: *P* = 0.006, effect size = 0.14; 3 years: *P* = 0.08, effect size = 0.07) and higher Ig class-switched B cell fractions (2 years: *P* = 0.02, effect size = 0.12; 3 years: *P* = 0.07, effect size = 0.07; Fig. 3b). This suggests circulating immune fraction may serve as a potential cancer marker.

### Association of genetic ancestry with lymphocyte fraction

Beyond the effects of age and sex, genetic ancestry may influence immune infiltrate^26^ and leukocyte counts in blood^27^. While germline variants associated with the immune system may affect cancer outcomes^28^, most relevant GWAS studies use samples from non-cancer patients.

The 100KGP participants were grouped into super-populations defined from the 1000 Genomes Project^29^. Significant differences in circulating T cell fractions were observed among genetically inferred ancestry groups in both healthy and cancer cohorts, with genetic African ancestry showing significantly higher immune fractions (Fig. 3c). However, no significant genetic ancestry-based differences were found in tumor-infiltrating T cell fractions (Fig. 3d).

**Extended Data Fig. 7:**
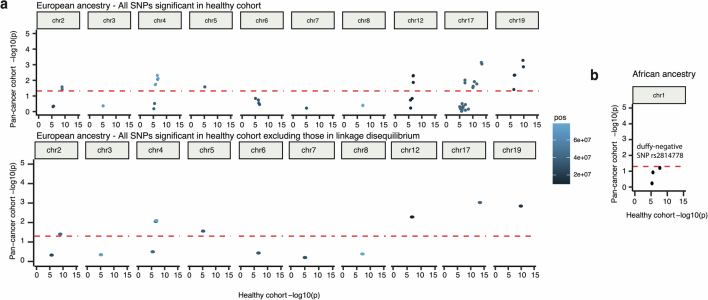
Additional determinants of ImmuneLENS fractions. **a**,**b**, Significance of hit SNPs from PLINK analysis for circulating *TCRA* T cell fraction in healthy cohorts identified in European ancestry (**a**) and African ancestry (**b**) versus their significance in the cancer cohort, with SNPs colored by their position within the chromosome to distinguish between multiple significant loci. Dashed red line represents p = 0.05 (unadjusted) in the cancer cohort. The P values are derived from the PLINK software that uses a linear regression model and performs a Wald test for each SNP. For the cancer cohort, this was done separately for each histology, and the P values were combined using a meta-analysis with a common effects model.

We examined 1,635 SNPs known to influence circulating leukocyte traits^27^ to evaluate whether these could explain differences in lymphocyte fractions between individuals (Methods and Supplementary Data). After accounting for linkage disequilibrium (LD), 15 SNPs were significantly associated with circulating T cell fraction in the healthy European cohort, but only one SNP in the European cancer cohort (Fig. 3e). The Duffy-negative SNP rs2814778, linked to neutropenia^30^, was significant in the African healthy cohort but not in the African cancer cohort (Fig. 3e). Only 7 out of 15 significant SNPs in the healthy European cohort were also associated with immune cell levels (unadjusted *P* < 0.05) in the European cancer cohort (Extended Data Fig. 7a,b). This suggests that germline SNPs influencing T cell fraction differ between healthy and cancer contexts (Supplementary Fig. 10).

### Quantifying selection pressure due to immune infiltration

We reasoned that the immune system may act as a potent selection pressure during cancer evolution. We, therefore, investigated whether mutations in known cancer genes were associated with tumor immune infiltrate.

**Fig. 4:**
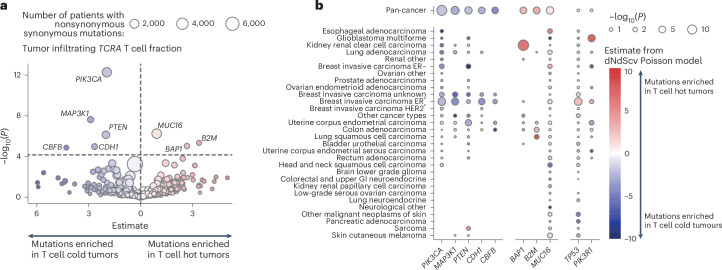
Association of selection with infiltrating T cell fraction. **a**, Volcano plot showing results from a Poisson model predicting observed nonsynonymous mutations as a function of *TCRA* T cell fraction and other covariates (age, tumor mutation burden, sex and disease type). The plot shows the estimates and −log_10_(*P*) for the *TCRA* variable, highlighting genes where observed mutations significantly depend on T cell immune infiltration (hot tumors denote high levels of immune cell infiltration; cold tumors lack immune cell infiltration). Point size represents the number of patients in the cancer cohort with nonsynonymous mutations, excluding patients with hematological cancers. Genes tested were limited to known cancer drivers from the Cancer Gene Census^47^. **b**, Bubble plot showing the significance of *TCRA* infiltrating T cell fraction within a Poisson model applied to individual cancer types. In both plots (**a**,**b**), *P* values represent the significance of the *TCRA* T cell fraction term in the Poisson model and are calculated using a Wald test.

**Extended Data Fig. 8:**
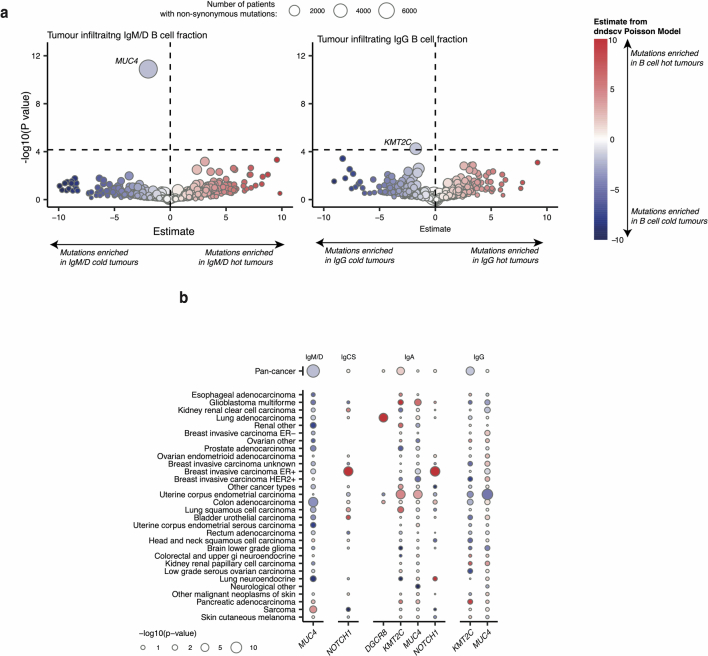
Association of selection with infiltrating B cell fraction. **a**, Volcano plots for the significance of association of IgM/IgD and IgG infiltrating B cell fraction for selection of nonsynonymous genes from the cancer gene census as measured with Poisson model: observed mutations ~ offset(log(expected mutation)) + age + sex + purity + tumor mutation burden + disease type + infiltrating B cells, with expected mutations calculated by dNdScv, and run on the entire pan-cancer cohort excluding hematological and childhood cancers. Dashed black lines are at estimate = 0, and the FDR significance threshold of −log_10_(P) = 4.16. **b**, Bubble plot showing disease-type-specific significance from Poisson model for different infiltrating B cell fractions, with genes selected as those that are significant either at the pan-cancer level (*MUC4* in IgM/IgD and *KMT2C* in IgG) or within a single disease-type at an adjusted P < 0.05 with genes only tested if 10 or more patients had nonsynonymous mutations within that gene in that cancer type. P values in **a** and **b** represent the significance of the term for the *TCRA* T cell fraction variable in the Poisson model and are calculated using a Wald test.

Using a Poisson model^31^, controlling for the background mutation rate, cancer type, sex and tumor purity, we identified seven genes significantly associated with infiltrating T cell fraction. Nonsynonymous mutations in *PIK3CA, MAP3K1, PTEN, CBFB* and *CDH1* were enriched in T cell-depleted tumors, while *MUC16, B2M* and *BAP1* mutations were enriched in T cell-replete tumors (Fig. 4a and Supplementary Data). Additionally, *MUC4* mutations were associated with IgM/IgD B cell-depleted tumors, while *KMT2C* mutations were linked to IgG B cell-enriched tumors (Extended Data Fig. 8a and Supplementary Data).

Disease-specific effects were also identified (Fig. 4b). For example, *TP53* nonsynonymous mutations were associated with increased T cell infiltrate in breast invasive carcinoma estrogen receptor-positive (BRCA ER^+^) tumors (estimate = 2.56, adjusted *P* = 6 × 10^−4^), while *PIK3R1* nonsynonymous mutations were associated with T cell-enriched glioblastomas (estimate = 7.23, adjusted *P* = 0.003). Previous studies have linked *TP53* alterations with increased T cell infiltrate in breast cancer^32^. Many disease-specific effects were also detected for B cell subsets. *MUC4* nonsynonymous mutations were significantly associated with reduced IgM/IgD B cells in colon adenocarcinoma (estimate = −3.7, adjusted *P* = 0.007). *NOTCH1* nonsynonymous mutations were associated with class-switched and IgA B cells in BRCA ER^+^ tumors (estimate = 10.6, adjusted *P* = 0.003), and nonsynonymous mutations in *DGRC8* were associated with enriched IgA B cells in lung adenocarcinoma (estimate = 18.7, adjusted *P* = 0.04). *MUC4* and *KMT2C* were both found to be significant in uterine corpus endometrial carcinoma, being associated with tumors enriched with IgA (*MUC4*: estimate = 3.58, adjusted *P* = 0.049; *KMT2C*: estimate = 4.80, adjusted *P* = 0.01) and depleted of IgG (*MUC4*: estimate = −4.85, adjusted *P* = 8 × 10^−5^; *KMT2C*: estimate = −3.82, *P* = 0.007, adjusted *P* = 1; Extended Data Fig. 8b and Supplementary Data).

These findings reveal diverse interactions between the tumor immune microenvironment and cancer cells. Somatic mutations may be selected in response to immune presence or promote immunosuppression, with effects varying by cancer type, histology and immune cell type.

### Prognostic value of ImmuneLENS lymphocyte fraction

Infiltrating tumor lymphocytes^6,33,34^ and circulating immune cells^10,35–38^ have prognostic value in many cancer types. However, a direct comparison of both has proven challenging. We therefore used ImmuneLENS to compare the prognostic significance of circulating and tumor-infiltrating T cell and B cell fractions.

**Fig. 5:**
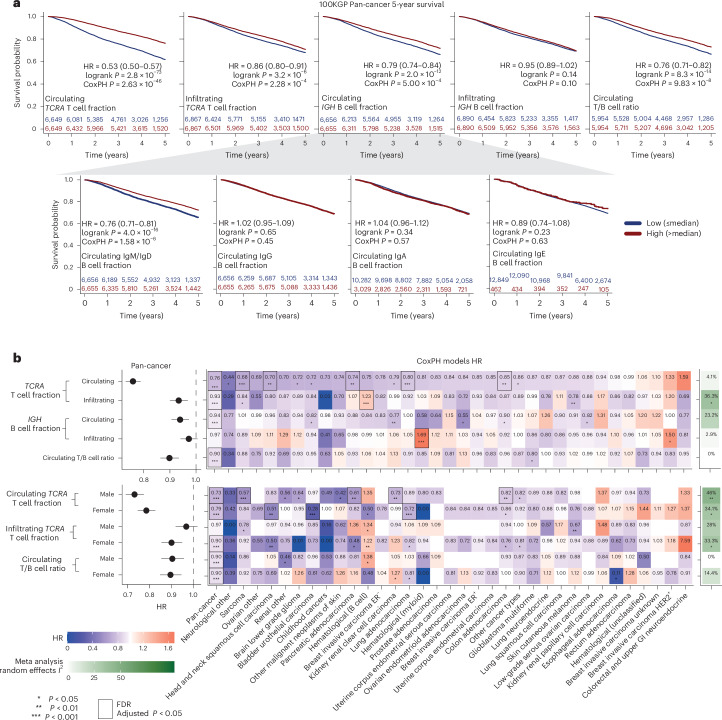
Prognostic value of ImmuneLENS lymphocyte fractions in 100KGP. **a**, Five-year survival Kaplan–Meier plots for the entire pan-cancer 100KGP cohort, stratified into high and low groups based on the median circulating or infiltrating *TCRA* T cell fractions and *IGH* B cell fractions. **b**, Results from CoxPH models for 13,872 participants within the 100KGP pan-cancer cohort with complete clinical annotation. The models account for the effects of age, sex, genetically inferred ancestry, pretreatment chemotherapy and cancer stage. Left, pan-cancer HRs with 95% CIs. Right, a heatmap of HRs for different cancer histologies, including the *I*^2^ score from a meta-analysis using a random effects model across all histologies. Significance was calculated using Cochran’s *Q* test. Multiple hypothesis adjustments were performed using the Benjamini–Hochberg method, applied by row. Individual *P* values were calculated using a two-sided Wald test within the Cox model. *P < 0.05, **P < 0.01 and ***P < 0.001.

In the pan-cancer cohort, elevated circulating T cell fractions were strongly associated with improved overall survival (Fig. 5a; hazard ratio (HR) = 0.53, log-rank *P* = 2.8 × 10^−73^, split by the median). Infiltrating tumor T cells showed a significantly weaker association (Fig. 5a; HR = 0.86, log-rank *P* = 3.2 × 10^−6^; *P* = 2.68 × 10^−^^25^, *z* test on the log of the HR values). Elevated circulating *IGH* B cells (HR = 0.79, *P* = 2 × 10^−12^) and IgM/IgD B cells (HR = 0.76, *P* = 4 × 10^−16^) were associated with better prognosis while infiltrating B cells and class-switched circulating B cells were not significant. The circulating T/B cell ratio, which is independent of neutrophil levels, was also significantly prognostic (Fig. 5a; HR = 0.76, log-rank *P* = 8.3 × 10^−14^).

**Extended Data Fig. 9:**
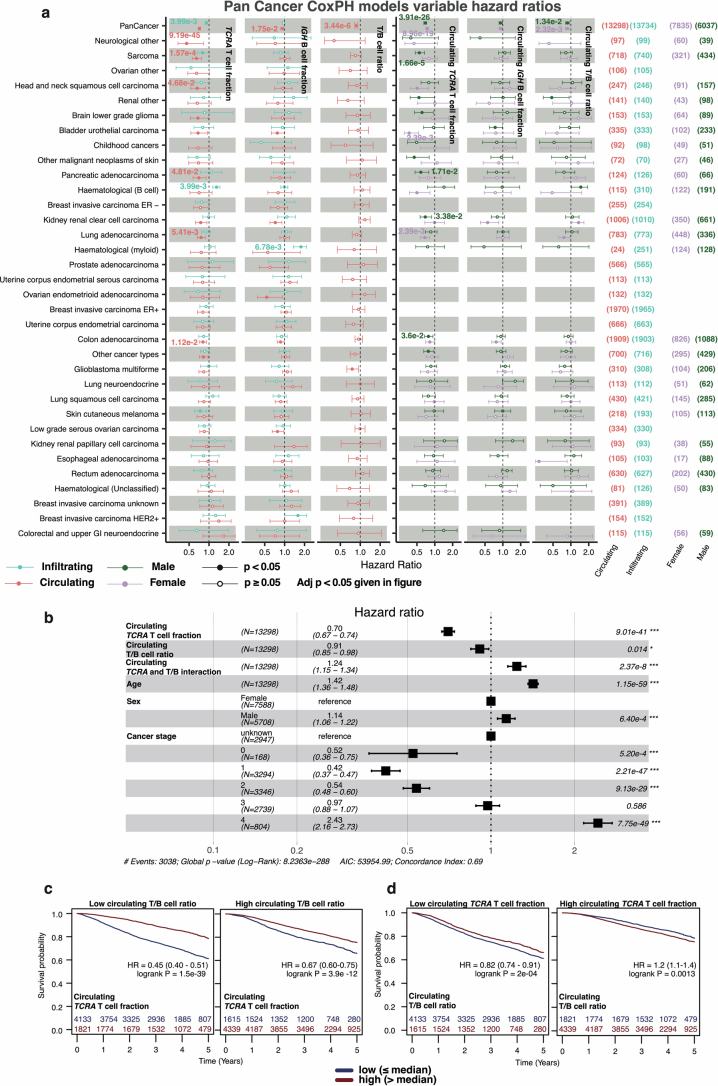
Survival forest plots and circulating T cell fraction and T/B cell ratio interaction. **a**, Output from CoxPH models showing hazard ratio with 95% confidence intervals for 5-year survival controlled for age, sex, cancer stage and treatment before surgery. Dashed black line represents a hazard ratio of 1. **b**, Results from a CoxPH model for circulating TCRA T cell fraction and T/B cell ratio together with their interaction term from the 100KGP pan-cancer cohort showing hazard ratio with 95% confidence interval. Dashed black line represents a hazard ratio of 1. **c**,**d**, Kaplan–Meier curves for 5-year survival for 100KGP pan-cancer cohort separated into either low- or high-circulating T/B cell ratio or circulating TCRA T cell fraction based on the median values. Multiple hypothesis adjustments were performed using the Benjamini–Hochberg method with individual P values calculated using a two-sided Wald test within the Cox model.

**Extended Data Fig. 10:**
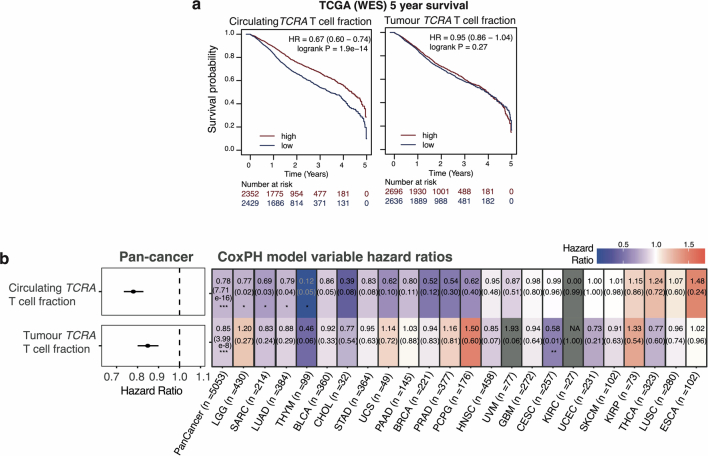
Survival analysis in TCGA. **a**, Five-year survival Kaplan–Meier curves for the TCGA data using the median value of *TCRA* T cell fraction as calculated by T cell ExTRECT to assign high and low groups. **b**. Left: hazard ratio associated with pan-cancer TCGA cohort with 95% confidence interval. Right: heatmap of hazard ratios for each individual cancer type with P values given in brackets. All P values were calculated using a two-sided Wald test within the Cox model. *, P < 0.05; **, P < 0.01; ***, P < 0.001. LGG, brain lower grade glioma; SARC, sarcoma; LUAD, lung adenocarcinoma; THYM, thymoma; BLCA, bladder urothelial carcinoma; CHOL, cholangiocarcinoma; STAD, stomach adenocarcinoma; UCS, uterine carcinosarcoma; PAAD, pancreatic adenocarcinoma; BRCA, breast invasive carcinoma; PRAD, prostate adenocarcinoma; PCPG, pheochromocytoma and paraganglioma; HNSC, head and neck squamous cell carcinoma; UVM, uveal melanoma; GBM, glioblastoma multiforme; CESC, cervical squamous cell carcinoma and endocervical adenocarcinoma; KIRC, kidney renal clear cell carcinoma; UCEC, uterine corpus endometrial carcinoma; SKCM, skin cutaneous melanoma; KIRP, kidney renal papillary cell carcinoma; LUSC, lung squamous cell carcinoma; ESCA, esophageal carcinoma.

Circulating T cell fraction remained highly significant after adjusting for clinical factors in the pan-cancer cohort (Fig. 5b (HR = 0.76, *P* = 2.6 × 10^−46^) and Extended Data Fig. 9a) and within five individual cancer types after multiple hypothesis correction—head and neck squamous cell carcinoma (HR = 0.70, adjusted *P* = 4.68 × 10^−2^), pancreatic adenocarcinoma (HR = 0.74, adjusted *P* = 4.81 × 10^−2^), colon adenocarcinoma (HR = 0.85, adjusted *P* = 1.12 × 10^−^^2^), lung adenocarcinoma (HR = 0.80, adjusted *P* = 5.4 × 10^−3^) and sarcoma (HR = 0.68, adjusted *P* = 1.57 × 10^−4^). Conversely, tumor-infiltrating T cell fraction was only significant in the pan-cancer cohort (HR = 0.93, adjusted *P* = 3.99 × 10^−3^) and B cell hematological cancers, where higher infiltration correlated with worse prognosis (HR = 1.24, adjusted *P* = 3.99 × 10^−3^). We observed broadly consistent results using TCGA data (Extended Data Fig. 10a,b). Moreover, we observed that the infiltrating T cell fraction was associated with significant heterogeneity by cancer type (*P* = 0.02, Cochran’s *Q* test).

Circulating T/B cell ratio remained significantly associated with survival after controlling for clinical variables (HR = 0.90, adjusted *P* = 3.4 × 10^−6^), suggesting that both circulating T cells and neutrophils contribute to the prognostic value of circulating T cell fraction. A Cox proportional hazard (CoxPH) model including circulating T cell fraction, T/B cell ratio and their interaction revealed independent significance for both factors (*TCRA*: HR = 0.7, *P* = 9 × 10^−41^; T/B ratio: HR = 0.91, *P* = 0.014; interaction term: HR = 1.24, *P* = 2 × 10^−8^; Extended Data Fig. 9b). When stratifying patients by T/B cell ratio, circulating T cell fraction was prognostic in both the low (HR = 0.45, 95% confidence interval (CI): 0.40–0.51; Extended Data Fig. 9c) and high T/B cell ratio group (HR = 0.67, 95% CI: 0.60–0.75; Extended Data Fig. 9c), but was significantly more prognostic in the low group (*P* = 2.4 × 10^−6^, *z* test). When stratifying patients by circulating T cell fraction, higher T/B ratio correlated with better outcome in the low T cell group (HR = 0.82, *P* = 2 × 10^−4^; Extended Data Fig. 9d), while the opposite was true for the high T cell group (HR = 1.2, *P* = 0.0013; Extended Data Fig. 9d). Thus, this suggests that in patients with relatively low neutrophil levels in their blood, B cells confer an improved prognosis compared to T cells.

We next investigated sex-specific prognostic associations of circulating T cell fraction (Fig. 5b). In the pan-cancer context, both male (HR = 0.73, *P* = 1.5 × 10^−27^) and female (HR = 0.79, *P* = 2.6 × 10^−20^) individuals showed similar associations between high T cell fraction and improved prognosis. However, cancer-specific differences were evident. For example, higher T cell fraction was associated with a significantly better prognosis for females in bladder urothelial carcinoma (HR = 0.28, adjusted *P* = 2.53 × 10^−3^) and lung adenocarcinoma (HR = 0.72, adjusted *P* = 2.53 × 10^−3^). A random effects meta-analysis showed circulating T cell fraction had a uniform prognostic effect across cancer types (*I*^2^ = 4%, not significant). However, sex-specific analysis revealed significant heterogeneity (male: *I*^2^ = 46%, *P* = 0.006; female: *I*^2^ = 34%, *P* = 0.03).

Considered together, these findings highlight the clinical importance of circulating lymphocytes and the interplay between biological sex and immune activation in different cancers.

## Discussion

We introduce ImmuneLENS, a method for inferring immune cell fractions from WGS data. Building upon our previous method T cell ExTRECT, ImmuneLENS not only provides more accurate T cell content inference but also expands functionality to measure B cell fraction, B cell class switching and T cell clonotype diversity.

To evaluate this approach, we applied it to the 100KGP cohort. In patients with cancer, circulating T cell and B cell fractions were reduced compared to healthy controls, and female individuals exhibited higher T cell fractions than male individuals, indicating significant sexual dimorphism. Although healthy controls also show sexual dimorphism, it appears mainly after age 55. Thus, ImmuneLENS quantified the sexual dimorphism in circulating immune cells, which is known to occur in both aging^39^ and cancer^40^.

In the healthy control cohort, we found significant associations between circulating T cell fractions and 15 SNPs at 11 genetic loci. This is consistent with recent work discussed in ref. ^41^, in which T cell ExTRECT^6^ was used on WGS data from 207,000 individuals and 27 loci associated with circulating T cell fraction were identified. Our analysis also identified an SNP within *FOXP1* (rs35592432; *P* = 7.3 × 10^−^^6^) that was not reported in ref. ^41^, with the variant allele associated with an enrichment of T cells. We observed that more than half of SNPs (8/15) linked to circulating T cell fractions in healthy individuals were not linked within the cancer population. This discrepancy highlights the need for further research on how germline genetics influence immune composition in cancer.

We found that circulating T cell fractions in patients with cancer are more prognostic than tumor-infiltrating T cells. This relationship may reflect systemic inflammation from tumor growth^42^. The association of the T/B cell ratio with prognosis suggests lymphocyte depletion, not just increased neutrophils, contributes to this signal. Circulating lymphocyte fractions may indicate ‘immunological age’^43^, reflecting the diminishing ability of the immune system to suppress cancer. This hypothesis is supported by the reduction we observed in circulating IgM/IgD B cell fractions of healthy participants who later developed cancer; however, we found no similar association with T cell fractions.

Despite the technological advancement of ImmuneLENS, there are limitations. While ImmuneLENS can accurately estimate immune cell fractions at ≥5× WGS coverage, additional sequencing coverage is required for B cell germline copy number correction (>10×) and somatic copy number adjustment (>20×). Moreover, TCR repertoire analysis from WGS data lacks the accuracy to make it comparable to TCR-seq, and predicting exact clonotypes remains a challenge. We do not envisage this method replacing TCR-seq. Rather, it can provide orthogonal insight that was previously lacking in the TCR repertoire in samples with solely WGS. ImmuneLENS can accurately estimate B cell fractions and deconvolve the separate class-switched fractions. This has much potential outside the context of cancer, for instance, within autoimmunity research^44–46^.

In summary, with the growing size of population-level WGS datasets, we have provided a tool that can accurately quantify lymphocyte fraction without the need for additional data collection. We hope ImmuneLENS will enable a deeper exploration of immune dysregulation.

## Methods

### Statistical information

All statistical tests were performed in R 4.0.2. No statistical methods were used to predetermine the sample size. Tests involving correlations were done using stat_cor from the R package ggpubr (v0.6.0) with Spearman’s method, except for situations when we directly tested if there exists a linear relationship between two variables for which Pearson correlation was used. Tests involving comparisons of distributions were done using stat_compare_means using wilcox.test using either the unpaired option, performing a Wilcoxon rank-sum (Mann–Whitney *U*) test, or a paired Wilcoxon signed-rank test. Effect size values for the corresponding Wilcoxon tests were measured using the wilcox_effsize function from the rstatix package (v0.7.2). HR values and *P* values were calculated with the survival package (v3.1-12) for both Kaplan–Meier curves and the CoxPH model. HR values between different models were compared by using a *z* test on the log of the HR values. For all statistical tests, the number of data points included are plotted or annotated in the corresponding figure. Plotting and analysis in R also made use of the ggplot2 (v3.4.1), dplyr (v1.1.0), tidyr (v1.3.0), gridExtra (v2.3), tidyverse (1.3.2), gtable (v0.3.2), scales (v1.2.1), lubridate (v1.9.2), survminer (0.4.9), survcomp (1.40.0), RColorBrewer (v1.1.3), GGgally (v2.1.2), ggforce (v0.4.1), TCellExTRECT (v1.0.1), MatchIt (v4.5.0) and dNdScv (v0.0.1.0) packages. *P* value adjustments were made using either the Holm–Bonferroni method or the false discovery rate (FDR)/Benjamini–Hochberg method, with the FDR method being used for exploratory analysis and those involving many tests.

### TRACERx 100

The TRACERx study (Clinicaltrials.gov registration: NCT01888601) is sponsored by University College London (UCL/12/0279) and has been approved by an independent Research Ethics Committee (13/LO/1546). All TRACERx samples used in this sample have been previously described^13^, and obtaining informed consent from each patient was a mandatory requirement for participation in the TRACERx study. Both WES (aligned to the hg19 sequence) and RNA-seq samples were obtained from the TRACERx study for the first 100 patients; the method for processing these samples is as previously described^13,48^. For the WES samples, exome capture was performed using a custom version of the Agilent Human All Exome V5 kit according to the manufacturer’s instructions.

TCR-seq TRACERx100 data used in this analysis have been previously published^49^; FASTQ data are deposited at the Sequence Read Archive (SRA) under accession code BioProject (PRJNA544699).

TRACERx100 DNA samples were sequenced and aligned to GRCh38 by Genomics England using the same Illumina sequencing pipeline as used in the 100KGP to produce WGS samples to a mean depth of 175× (median 223×). WGS coverage values from the *TCRA, TCRB, TCRG* and *IGH* loci for these samples were then extracted for use for ImmuneLENS using SAMtools (v.1.3.1) depth. No other WGS-derived data besides these coverage values was used from the TRACERx cohort for this analysis.

### 100KGP WGS cohort

The 100KGP was ethically approved by the East of England—Cambridge South Research Ethics Committee (Research Ethics Committee reference 14/EE/1112, Integrated Research Application System ID 166046). Participants were recruited from 13 National Health Service (NHS) Genomic Medicine Centres, and all provided written informed consent.

All WGS samples within the 100KGP cohort were sequenced by Illumina and carried out on behalf of Genomics England. The Illumina pipeline for all samples used in this analysis performed alignment with the Issac aligner to the GRCh38 reference. Full details can be found at https://re-docs.genomicsengland.co.uk/genomic_data. Tumor samples were sequenced to a median depth of 97.5×, while germline blood samples were sequenced to a median depth of 32.7× in the pan-cancer cohort and 39.7× in the rare disease cohort.

T cells and B cells were calculated for the entire 100KGP cohort (lung—data release v8 (28 November 2019), remaining pan-cancer data release v12 (06 May 2021) and rare disease v12 (07 May 2021)). In total, scores were calculated for 92,905 WGS BAM files.

Of these, 31,675 BAM files were part of the 100KGP cancer cohort, representing 16,294 cancer BAM files and 15,381 germline BAM files. Some participants had multiple tumor samples collected for WGS; due to the lack of annotation of the reason for multiple samples (for example, technical resequencing, representative of metastasis, multiple region sequenced or occurrence of a second primary tumor at a later time point), these were removed from the pan-cancer cohort, leading to a final cohort of 14,501 tumor WGS samples. Of the 14,501 tumor WGS samples of our cohort, 13,868 have a matched blood WGS sample.

For the rare disease cohort, scores for 61,230 BAMs in total were calculated. Limiting to samples taken from blood samples leads to 59,903 BAMs representing 29,238 samples taken from probands with a rare disease and 30,665 relatives of these probands. This cohort of 30,665 blood samples from relatives was taken as our healthy cohort to compare with the 13,868 blood samples from patients with cancer. Additionally, from this healthy cohort, propensity-matched cohorts for each cancer histology were created using the R package matchit, controlling for both age and sex.

T cell and B cell fractions were calculated with the WGS version of ImmuneLENS; adjustments for tumor purity were made using estimates from Genomics England and local copy number using CANVAS (v.1.3.1) calls produced by Genomics England for nearby genes to the V(D)J loci (*TCRA*—*OR10G3*; *TCRB*—*PRSS58*; *TCRG*—*STARD3NL*; *IGH*—*TMEM121*).

Cancer histology in terms of disease and disease subtype was curated by Genomics England and is as described in the cancer_analysis_table available to researchers within the Genomics England research environment. To be consistent with other pan-cancer analyses, particularly TCGA, we used the histology groups designed by Genomics England to align as closely as possible to the TCGA (https://re-docs.genomicsengland.co.uk/cancer_analysis_histology/). We, however, kept the Childhood Cancers group from Genomics England’s own annotation of cancer disease type due to the effect of age on our analysis. We also split up the breast invasive carcinoma group by hormone receptor status where available, and for the hematologically derived cancers, we split them up by cell of origin to distinguish B cell- and T cell-derived cancers from those from myeloid cells. All cancer types with occurrences less than 100 cases in the total cohort were assigned to the other cancer type groups, for both ease of analysis and to avoid the risk of any personally identifying features.

### TCGA pan-cancer data

T cell ExTRECT was applied to the pan-cancer TCGA WES dataset. For different TCGA cohorts, different exome capture kits were used, and exon quality control was undertaken to ensure consistent results across the entire TCGA cohort. In brief, for each capture kit, the median GC-corrected read depth ratio was calculated for each exon using the exonsTcellExTRECT function across all samples. Exons with low coverage (median read depth ratio < −0.5) were filtered from the capture kit BED file.

TCGA sample ancestry calls are the consensus of five genetic ancestry calling approaches described in ref. ^50^.

### 1000 Genome cohort

In total, 2,544 samples with matched high- and low-coverage CRAM files, along with their indexed CRAI files, were downloaded directly from the 1000 Genomes cohort server (ftp://ftp.1000genomes.ebi.ac.uk) using wget. The median depth of the high-coverage samples was 34×, and the median depth for the low-coverage samples was 1.25×. ImmuneLENS with SAMtools (v.1.3.1) was then used on these CRAM files to extract the coverage and then calculate T cell and B cell fractions. Processed RNA-seq data from the Geuvadis project for 465 lymphoblastoid cell lines from the 1000 Genomes were downloaded from https://www.ebi.ac.uk/gxa/experiments/E-GEUV-1/Downloads with RNA-seq analysis performed using R packages limma and edgeR.

### PCAWG

Our analysis was restricted to the TCGA portion of the PCAWG that contained 539 WGS tumor-normal pairs from BAM files that had been realigned to hg38 at MD Anderson. Germline normal samples had a median depth = 37.3× and tumor samples median depth = 51.2×. ImmuneLENS/SAMtools (v.1.3.1) was used to extract coverage files for the *IGH* and *TCRA* loci, which were then used in the calculation of all T cell and B cell fractions using the ImmuneLENS R package.

### Low-pass TCGA data

In total, 317 low-pass TCGA BAM files with a median depth of 4.95× that were from samples with corresponding PCAWG high-coverage WGS were downloaded using the TCGA GDC client; coverage values for each sample were downloaded from the GDC client API.

### scRNA cohort and analysis

Processed scRNA data with associated metadata from a lung cancer dataset described in ref. ^51^ were used. Annotation of B cell subtypes was used, and class switching was determined from the expression of *IGH* class switch segments with cells with unclear annotation removed from the analysis.

### Nested downsampling of WGS files

Nested downsampling was performed on WGS BAM files using SAMtools view (v.1.3.1) recursively with the following options:

samtools view -b -h --subsample FRAC - -subsample-seed SEED BAM CHR_LOC > OUT_BAM

The depth of the original BAM files was calculated with mosdepth (v.0.3.2) and then downsampled to 60×. After each downsampling, each output BAM was indexed using Picard (v.2.20.3) BuildBamIndex before being downsampled again with SAMtools view to create a set of nested downsampled BAMs with depths of 60, 50, 40, 30, 20, 10, 5, 2, 1, 0.5 and 0.1×. The values for FRAC were calculated to obtain these depths based on the depth of the original BAM file as calculated with mosdepth. SEED values were changed in each nested down sample to avoid using the same seed repeatedly.

The above-given procedure was done to generate downsampled BAMs for all of the TRACERx WGS samples for the *TCRA* (chr14: 21621904–22752132), *TCRB* (chr7: 142299011–142813287), *TCRG* (chr7: 38240024–38368055) and *IGH* (chr14: 105566277–106879844) loci. These downsampled BAMs were then used as input for the WGS version of ImmuneLENS to calculate the T cell and B cell fractions.

### Differential gene expression analysis and gene set enrichment

We performed differential gene expression on TRACERx100 patients with RNA-seq data, separating the cohort into high or low groups based on either *IGH* B cell fraction or class-switching B cell fraction scores for *IGHG1, IGHA1* B cell fraction or nonclass-switched IgM/IgD B cell fraction. First, using R 4.0.0, the edgeR package (v.3.32.1) was used for the sample-specific trimmed mean of the *M* values normalization; any genes with low expression were then filtered out using the standard edgeR filtering method before using the limma–voom method from the limma R package (v.3.46.0) to calculate the voom fit and obtain *P* values for the gene-expression differences. The comparison controlled for patient and histology as blocking factors, and *P* values were FDR-corrected for multiple testing. Results were then visualized with the R EnhancedVolcano package (v.1.8.0). Gene set enrichment analysis was then performed using the fgsea R package, which uses (v.1.24.0) the MSigDB C8 genesets of cell type signatures (https://www.gsea-msigdb.org/gsea/msigdb/human/genesets.jsp?collection=C8). An adaptive multi-level split Monte Carlo scheme for *P* value estimation was used and full results are given in Supplementary Data.

### Calculation of TCR/BCR diversity metrics

T cell diversity metrics from V or J segment usage were predicted by the ImmuneLENS model. For the Shannon diversity, we used the formula:

$$\mathrm{Shannon}\,\mathrm{diversity}=-\sum _{i}{p}_{i}ln(\,{p}_{i}),$$

where ${p}_{i}$ represents the proportion of an individual V or J segment predicted from the model.

To compare between two samples with different predicted segment usage, we used the Jensen–Shannon divergence metric defined as follows:

$$P=\mathrm{probability}\,\mathrm{distribution}\,\mathrm{of}\,\mathrm{V}\,\mathrm{or}\,\mathrm{J}\,\mathrm{segment}\,\mathrm{usage}\,\mathrm{in}\,\mathrm{sample}\,\mathrm{A}.$$

$$\mathit{Q}=\mathrm{probability}\,\mathrm{distribution}\,\mathrm{of}\,\mathrm{V}\,\mathrm{or}\,\mathrm{J}\,\mathrm{segment}\,\mathrm{usage}\,\mathrm{in}\,\mathrm{sample}\,\mathrm{B}.$$

$$M\,=\,\frac{P\,+\,Q}{2}.$$

$D({P|Q})\,={\sum }_{i}{P}_{i}\,\log (\frac{{P}_{i}}{{Q}_{i}})$, where ${P}_{i}$ and ${Q}_{i}$ represent the proportion of segment *i* used in sample A or B, respectively.

The Jensen–Shannon divergence (JSD) is then defined as follows:

$${\mathrm{JSD}}({P|Q})\,=\,\frac{1}{2}D({P|M})+\,\frac{1}{2}\,D({Q|M}\,).$$

MiXCR^22^ was used to call TCR clonotypes from TRACERx 100 RNA-seq data directly. From these calls, the Shannon entropy was then calculated using the proportions of the TCR clonotypes found in each sample.

TRUST4 (ref. ^52^) was also used to call BCR sequences and therefore infer class-switching proportions.

### TRAV segment usage analysis

TRAV segment predictions from ImmuneLENS were generated for the entire 100KGP cohort. For analysis of differing TRAV segment usage, we restricted to samples with >0.05 total *TCRA* T cell fraction. To test for TRAV segment usage changes between different populations, we used propensity matching to control for *TCRA* T cell fraction and to ensure that the distribution of T cell fraction was the same between comparison populations and hence there would be no bias with higher T cell fraction cohorts associated with more diversity due to our increased power to detect TRAV segment usage. Once the comparison cohorts were created, we defined a TRAV segment as being selected within the ImmuneLENS model if it had a fraction >0.001 of T cells predicted to use the TRAV segment. For every segment, we then calculated the percentage of samples within the cohort that selected that segment >0.001 fraction. Differences in segment usage between cohorts were then assessed using a *χ*^2^ test, and then *P* values were adjusted for multiple hypothesis testing.

### 100KGP genetic ancestry inference

Genetic ancestry inference was provided by Genomics England for the entire 100KGP cohort using ethnicities from the 1000 Genomes Project phase 3 as truth by first generating principal components and then projecting the 100KGP project onto them to identify the broad genetic ancestry super-category of each participant. Full details can be found at https://re-docs.genomicsengland.co.uk/ancestry_inference/.

### 100KGP date-matched blood count data

Blood count data were only available for a subset of the rare disease cohort within the 100KGP. We selected blood count data that were time-matched for the exact date of genomic sample collection resulting in data from 441 participants for which we had date-matched blood count and calculated T cell or B cell fractions. From this data, we further subsetted for participants with matched albumin count (*n* = 361), lymphocyte count (*n* = 222), neutrophil count (*n* = 84) and both neutrophil and lymphocyte count data (*n* = 84).

### 100KGP treatment data

Treatment data were extracted from 100KGP using the clinical data available in the Genomics England research environment from the ‘cancer_systemic_anti_cancer_therapy’ version 13 table.

### dNdScv analysis of selection in protein-coding genes

We used dNdScv^31^ to measure the expected number of nonsynonymous mutations for genes associated with cancer within the cancer gene census. We then adapted code from ref. ^53^, which used a Poisson model of observed nonsynonymous mutations using the neutral background expectation as calculated within dNdScv as an offset variable influenced by age or sex in normal bladder tissue. In our analysis, we also added tumor purity, tumor mutation burden and disease type as controlling variables. We added either infiltrating *TCRA* T cell fraction, *IGH* B cell fraction, total class-switched B cell fraction, total non-class-switched B cell fraction (IgM/IgD), IgA or IgG B cell fraction into the Poisson model and identified genes for which these variables were significant (model: observed mutations ~ offset(log(expected mutations)) + age + tumor mutation burden + sex + disease type + immune fraction). For the pan-cancer analysis, we selected significant genes after adjusting for multiple hypothesis testing, for the disease-type-specific models, we only tested genes that had ≥10 tumors containing nonsynonymous mutations in that disease type and did not include sex in the model for cancer types predominant in one sex.

### Analysis of known SNPs associated with leukocyte traits using PLINK

In total, 1,962 SNPs known to be associated with leukocyte traits (either basophil, eosinophil, lymphocyte, monocyte, neutrophil counts or white blood count) were downloaded from the data released by ref. ^27^. Of these, only 1,635 SNPs were listed within the VCF files provided within the 100KGP above the 0.001 mean allele frequency threshold. PLINK was used to test for associations between these SNPs and *TCRA* T cell fraction, *IGH* B cell fraction, IgM/IgD B cell fraction, IgG B cell fraction, IgA B cell fraction or the T/B cell ratio. All T cell and B cell fractions were first transformed using the inverse normal transformation to ensure normality. Association tests were run separately in the different genetic ancestry groups (as defined by the 1000 Genomes Project). Our analysis focused on participants with genetically inferred European ancestry, as this was the largest ancestry group in both the healthy and pan-cancer cohorts and those with genetically inferred African ancestry, as this group showed the most significant difference in circulating T cell fraction compared to the European ancestry group. For the healthy and pan-cancer cohorts age, sex and the first ten genetic ancestry principal components (PCs) were used as covariates. PLINK (v1.9) was run separately on each cancer subtype using these covariates. PLINK was run with the following steps: (1) LD pruning of the tested SNPs using the option –indep-pairwise 500 5 0.5 to test 500 SNPs at a time, moving the window by five SNPs at each step and using an *R*^2^ threshold of 0.5 to remove any high LD SNPs. (2) A cutoff on genotype frequencies using –geno 0.2 –maf 0.01 to remove any SNPs with a missing genotype rate >20% and a minor allele frequency >1%. (3) A Hardy–Weinberg filter using –hwe 0.000001 to specify the *P* value threshold. (4) A test for association of the phenotype using linear regression and the –linear option, as well as calculation of genotype frequencies using –freq. For the pan-cancer analysis, the output of each cohort from PLINK was then combined in a common effect meta-analysis using the metagen function from the R package meta, with the input being the treatment effect and its s.e. from each of the separate cancer histology runs of PLINK.

### Survival analysis

Survival data were collated on the Genomics England research environment using available data for date of cancer diagnosis, death records from the Office of National Statistics and the latest follow-up times from the most recent records in the hospital episode statistics (release v16). The data then underwent additional quality control to exclude any patients with any conflicting data, such as follow-up times greater than 10 years resulting from multiple dates of diagnosis values from previous incidences of cancer. In total, 13,348 participants with both survival data and ImmuneLENS fractions in blood and 13,342 participants with both survival data and ImmuneLENS fractions in tumor tissue were available for analysis.

A meta-analysis using a random effects model was applied to the output of the HRs of the disease-specific CoxPH models using the R function metagen from the package meta (v.6.5-0). Thus, the *I*^2^ values were calculated, and their significance was tested using Cochran’s *Q* test.

### Figure generation

Biorender.com aided in the generation of Fig. 1a, Extended Data Fig. 4a and Supplementary Fig. 3a.

### Reporting summary

Further information on research design is available in the Nature Portfolio Reporting Summary linked to this article.

## Online content

Any methods, additional references, Nature Portfolio reporting summaries, source data, extended data, supplementary information, acknowledgements, peer review information; details of author contributions and competing interests; and statements of data and code availability are available at 10.1038/s41588-025-02086-5.

## Supplementary information

Supplementary Information Supplementary Note (description of ImmuneLENS model, additional validation and analysis) and Supplementary Figs. 1–10.

Reporting Summary

Supplementary Data Clinical data for the 100KGP cohort, raw output for the TRACERx 100 limma analysis and output of the 100KGP analysis, including correlations between circulating and infiltrating immune scores, differences in TRAV segment usage, sexual dimorphism and propensity matching between cancer and normal analysis, complete CoxPH model outputs, output from the PLINK analysis of the ref. ^27^. SNP set and results from the Poisson dNdScv selection analysis.

## Data Availability

The RNA-seq and WES data (in each case from the TRACERx study) used during this study are a subset of the TRACERx421 dataset and have been deposited at the European Genome–Phenome Archive, which is hosted by The European Bioinformatics Institute and the Centre for Genomic Regulation under the accession codes EGAS00001006517 (RNA-seq) and EGAS00001006494 (WES). Access is controlled by the TRACERx data access committee to ensure patient privacy and data confidentiality are protected while fostering impactful scientific discoveries. Details on how to apply for access are available on the linked pages. The data access committee aims to respond to requests within 1 week. For TCR-seq data used in this analysis, the FASTQ data are deposited at the SRA under accession code BioProject (PRJNA544699). Coverage files for the *TCRA*, *TCRB*, *TCRG* and *IGH* loci were generated from WGS TRACERx 100 samples. These coverage files used for the calculation of the T cell and B cell fractions are available at Zenodo (10.5281/zenodo.7785803)^54^ and were the only data derived from the TRACERx WGS analysis used within this paper. WGS and phenotypic data from the 100KGP can be accessed by application to Genomics England following the procedure outlined at https://www.genomicsengland.co.uk/join-us for both academic and industry users. Genomics England restricts access to 100KGP data to bona fide researchers to protect the sensitive genomic data of its participants. For academic users, Genomics England aims to review all applications within ten working days, and access will be granted within two working days after confirmation of affiliation from the researcher’s institution and completion of online governance training. The 1000 Genome Data used are publicly available and can be accessed at https://www.internationalgenome.org/data. Calculated ImmuneLENS output including class-switching and polyclonal predictions for each LCL cell line included are available on Zenodo (10.5281/zenodo.11093976)^55^. PCAWG data used in this study were obtained through our collaboration with MD Anderson. To gain access to the raw WGS samples of the TCGA portion of the PCAWG data used in this study, researchers need to apply to the TCGA data access committee via a database of Genotypes and Phenotypes (dbGaP; https://dbgap.ncbi.nlm.nih.gov/aa/wga.cgi?page=login). Access is controlled due to respect and protection of the interests of research participants. The calculated ImmuneLENS output for these samples is available on Zenodo (10.5281/zenodo.11093961)^56^. TCGA pilot project was established by the National Cancer Institute (NCI) and the National Human Genome Research Institute. The data were retrieved through the dbGaP authorization (accession phs000178.v9.p8). Information about TCGA and the constituent investigators and institutions of the TCGA research network can be found at http://cancergenome.nih.gov/. To access TCGA WES and low-pass WGS data, researchers will need to apply to the TCGA Data Access Committee (DAC) via dbGaP (https://dbgap.ncbi.nlm.nih.gov/aa/wga.cgi?page=login). Access is controlled due to respect and protection of the interests of research participants. The calculated T cell ExTRECT *TCRA* T cell fraction scores along with the ImmuneLENS output for the low-pass WGS samples used in this study are available at Zenodo (10.5281/zenodo.7794867)^57^. Single-cell data used in this analysis are previously described^51^ and available at https://cellxgene.cziscience.com/collections/edb893ee-4066-4128-9aec-5eb2b03f8287.

## Code availability

The code used to produce T cell and B cell fraction scores will be made available for academic noncommercial research purposes as the R package ImmuneLENS, available for download and installation at https://github.com/McGranahanLab/ImmuneLENS.

All other code used in the analysis and necessary data to reproduce figures is available at Zenodo^58^ (10.5281/zenodo.14046632). 100KGP data cannot be exported outside the Genomics England research environment. All data and code to reproduce the 100KGP analysis, including T cell and B cell fractions, are available within the Genomics England research environment (see https://re-docs.genomicsengland.co.uk/access/ for information on using the research environment) within the folder ‘/re_gecip/shared_allGeCIPs/rbentham/ImmuneLENS_figure_code/’. Researchers can gain access to the Genomics England research environment and associated data following application to Genomics England following the procedure outlined at https://www.genomicsengland.co.uk/join-us.
