# Supplementary material for: ImmuneLENS characterizes systemic immune dysregulation in aging and cancer
Source: Nat Genet. 2025 Feb 18;57(3):694–705. doi: 10.1038/s41588-025-02086-5 (PMC11906351; doi:10.1038/s41588-025-02086-5)
Supplement: Supplementary file 1 — Supplementary Note (description of ImmuneLENS model, additional validation and analysis) and Supplementary Figs. 1–10. [file 41588_2025_2086_MOESM1_ESM.pdf]

nature genetics

Supplementary information https://doi.org/10.1038/s41588-025-02086-5

ImmuneLENS characterizes systemic immune dysregulation in aging and cancer

In the format provided by the authors and unedited

Supplementary Note

Supplementary Note 1

- Creation of a segment-based model to estimate immune cell fraction from whole genome sequencing (WGS) data 2
  - Description of ImmuneLENS model 2
  - Quality control of genomic loci within V(D)J genes used in ImmuneLENS 4
  - Germline and somatic IGH focal copy number correction 4
- Additional validation of ImmuneLENS 6
  - IGH germline and somatic copy number validation 6
  - Comparison of ImmuneLENS and T cell ExTRECT on TRACERx WGS vs WES 9
  - RNAseq validation of IGH class switching in the TRACERx100 cohort and scRNA data 10
  - IGH B cell fraction validation using the 1000 genomes cohort 12
  - Performance of ImmuneLENS on matched high and low depth WGS data sets 14
  - Use of ImmuneLENS in additional pan-cancer WGS data sets 16
- Extended analysis of 100KGP 18
  - ImmuneLENS reveals differences in TCR repertoire 18
  - Fold change analysis of circulating lymphocyte fractions between males and females in the healthy and cancer cohort 20
  - GWAS SNPs associated with circulating TCRA T and IGH B cell fraction 21
- Supplementary References 24

Creation of a segment-based model to estimate immune cell fraction from whole genome sequencing (WGS) data

Description of ImmuneLENS model

In T cell ExTRECT, we used a general additive model (GAM) to estimate r_{VDJ} — the read depth ratio at the maximum locus of V(D)J recombination — from whole exome sequencing (WES) data. We then calculated the T cell fraction from the detected depletion in r_{VDJ} [1]. Because standard exome capture kits do not sequence large parts of the TCRA locus—leading to non-uniform coverage in WES data—we modeled V(D)J recombination as a smoothed process. However, this simplification made the model more sensitive to noise and did not reflect the true biological process of V(D)J recombination.

In ImmuneLENS, we developed a model directly based on the biological process of V(D)J recombination. We fit the read depth ratio to constant piecewise segments, similar to ASCAT's[2] allele-specific copy number segmentation. However, unlike ASCAT, we know the precise locations of potential copy number breakpoints—representing the starts of deletion sites after V(D)J recombination—from the genomic positions of the V and J gene segments. Furthermore, the read depth ratio should start at 0 and decrease monotonically until the point of maximum V(D)J recombination. For example, in *TCRA* V(D)J recombination, only some TCR chains select the first *TRAV-1* segment; all other V segments are deleted. However, all TCR chains have a deletion after the final V segment. Similarly, for J segments, the read depth ratio increases monotonically to 0. This model could then be applied to the following V(D)J gene: *TCRA, TCRB* or *TCRG* for T cells.

To fit this model, we transformed each segment defined by V and J gene breakpoints into binary vectors, assigning 1s within their regions and 0s outside. Using a constrained linear model, we fitted these vectors to the normalized read ratios, applying the following inequality constraints for each of the *n* V and *m* J segments:

$V_1 < 0$

$V_2 < V_1$

...

$V_n < V_{n-1}$

$J_1 > V_n$

$J_2 > J_1$

...

$J_m > J_{m-1}$

$J_m < 0$

While fitting the V(D)J recombination model, we also adjusted for GC biases in the data by fitting values to the GC content within 100bp bins, their smoothed values in a vector and the square of these values. Using the fitted values from the model, we calculate the total T cell fraction from the model's maximum deviation, such as the value from the last V segment. We also determine individual fractions from specific segments.

To extend the model to the *IGH* locus for quantifying B cell fractions, we included segments representing class-switching deletion events that result in different antibody production. The breakpoints of these deletion events were restricted to their genomic locations—the coordinates of the S regions where class-switching breakpoints occur. The locations of the S regions were identified from the literature[3] and confirmed by detecting split reads within the 100KGP cohort. These split reads had one mate aligning upstream of the class-switch segment and the other downstream of *IGHM*. We found that the S region listed for *IGHG2* in Kuri-Magaña et al. better fit *IGHG4*, so we redefined the S regions based on the presence of split reads (coordinates in hg38: *IGHM:* chr14:105856501-105860500, *IGHG3*: chr14:105773001-105774000, *IGHG1*: chr14:105744501-105745500, *IGHA1*:chr14:105709501-105712500, *IGHG2*: chr14:105645000-105648000, *IGHG4*: chr14:105627501-105628500 , *IGHE*: chr14:105602501-105604500 and *IGHA2*: chr14:105589001-105591500, see Supplementary Fig. 1).

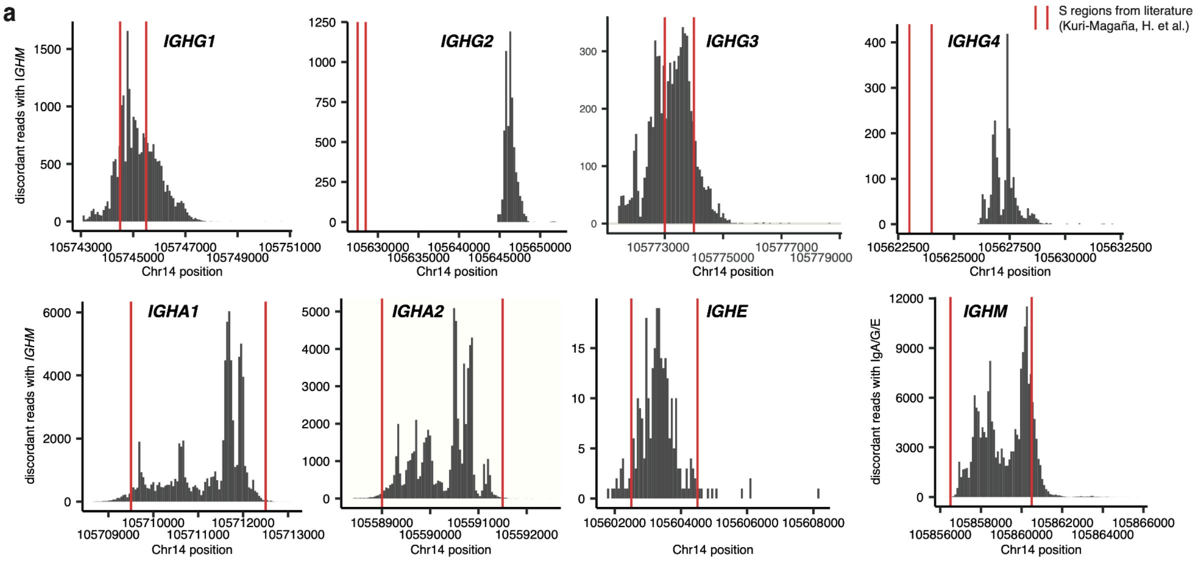

Supplementary Figure 1

a. Frequency of discordant split reads between around expected class switching S region between iG class switching segments and *IGHM*.

Within the model, we set the ends of the class-switching segments to the ends of the corresponding S regions (or the beginnings for *IGHM*). Because the exact breakpoint location within the S region is unknown, we masked coverage values from the ends of the class-switching segment exons and the ends of the S regions.

There were two additional restrictions for the IGH model:

1. The total fraction of class-switched B cells must be less than or equal to the total B cell fraction measured from the V(D)J region.
2. The final V segment, *IGHV3-73*, was restricted to be less than 0.01 times the total B cell fraction.

These restrictions prevent model solutions with artificially high B cell fractions and clonally dominant *IGHV3-73* segments, which were frequently observed due to overfitting of the GC correction terms. The fraction for non-class switched IgM/D B cells was calculated as the total B cell fraction minus the class switched B cell fraction.

Quality control of genomic loci within V(D)J genes used in ImmuneLENS

We performed a robust quality control process to identify 100bp segments in the V(D)J genes that had outlier coverage either (1) across the entire cohort or (2) within a subset linked to known germline genomic variants.

As the first step, we calculated the mean GC-corrected ratios in all the V(D)J genes across the 100KGP lung cancer cohort. This revealed 100bp segments with anomalously low or high coverage compared to surrounding regions. We fitted a GAM model to the mean GC-corrected ratios and flagged for exclusion any segments deviating from the fitted line by more than ±0.25. This approach worked well for *TCRA*, but for other genes, such as *TCRB*, large clustered segments interfered with the GAM model fit. For these genes, some clear outlier regions were initially removed manually—for example, all *TCRB* regions with a mean GC ratio above 0.25.

After removing these 100bp segments, we identified additional biased segments using a GWAS-type analysis with PLINK software. We found germline SNPs within the *TCRA/TCRB/TCRG/IGH* loci strongly associated with changes in ImmuneLENS-estimated T or B cell fractions. We then tested whether the 100bp segments containing these SNPs showed under- or overcoverage compared to surrounding regions, but only in patients with the alternative allele. This procedure identified additional outlier segments, which we flagged for removal. We reran the PLINK/GWAS analysis without the flagged regions to ensure no additional artifact segments remained.

Germline and somatic *IGH* focal copy number correction

To identify a patient's *IGH* locus copy number haplotype, we used germline blood samples assumed to have low B cell content (<10%). Next, we divided the GC-corrected coverage by the median read depth, smoothed it using a 1kb rolling average, and rounded to the nearest 0.5. We then categorized genomic regions as having loss or gain events. At each of five iterations, we recalculated GC-corrected coverage values, dividing by the median read depth only in regions without predicted copy number changes. After determining the *IGH* germline copy number variations (CNVs), we normalized the raw *IGH* coverage values. For regions with predicted loss events, we doubled the coverage values (if one allele was lost) or set them to zero (if deleted). For regions with predicted gains, we divided coverage values by the number of extra copies.

After this germline correction, we applied the ImmuneLENS method to calculate the IGH B cell fraction as described above

For somatic tumour samples, we ran a simplified copy number alteration (CNA) caller within the *IGH* locus using paired germline and tumour coverage values. We blacklisted the focal regions of expected class switching (hg38: chr14:105712500–105860500) and V(D)J recombination (hg38: chr14:105865458–105939756) to reduce the likelihood of these events being called as tumour somatic events. For somatic CNA calling, we first adjust both germline and tumour coverage values for GC content, then divide each by the median coverage within the IGH locus. We calculated the log ratio (logR) at each base as the $\log_2$ ratio of GC-adjusted tumour reads to germline reads, then normalized it by the median value in 1kb bins. We then grouped the logR values into line segments by fitting a recursive partitioning and regression tree using the rpart R package (version 4.1). For each segment, we calculated the median logR. We then perform a breakpoint check to see if any somatic segment breakpoints fall within the V(D)J or class-switching blacklisted regions. If so, we adjust the breakpoint to best fit the differences in $\log_2$ ratio between the segments on either side of the blacklisted region. We did this for every segment overlapping the blacklisted region with a logR value not equal to 1 (indicating a copy-number change overlapping the blacklisted region). We re-included the blacklisted regions and reran the recursive partitioning and regression tree on that overlapping segment, splitting it into subsegments. We then checked each new subsegment to see if its breakpoint matches the logR difference between the original overlapping segment and the adjacent one. If this difference is small (<20% difference between the logR change in the subsegments and the original adjacent segment), we choose it as the new breakpoint. After calling potential somatic CNAs, we only use them for normalization if the segments with predicted somatic CNA are >100 kb and the maximum logR change among all segments is >0.2. If these conditions are met, we correct the tumour coverage regions for the somatic CNA by dividing the coverage within this region by the ratio of the segment's median coverage to the baseline median coverage of the entire IGH locus.

After the somatic CNA correction, we correct for any germline copy number alterations identified in the matched germline sample. We adapt the germline correction process to account for tumour purity and ploidy. Instead of using theoretical normalization values valid for diploid tumors, we calculate the observed coverage ratio for each segment with called copy number alterations. This ratio compares the coverage in the tumor segment to a baseline from the entire *IGH* locus. These normalisation values are then used directly to perform germline correction for the tumour samples. We then calculate the *IGH* fractions from the tumour sample using both somatic and germline corrections, depending on whether the called somatic CNAs passed the QC checks (somatic CNA >100 kb and maximum logR change >0.1). Even if the somatic CNA correction passes QC, we also run the germline correction alone. As a final check, we accept the combined correction only if it is less than the germline correction alone. We do this because samples with confirmed somatic CNAs in the *IGH* locus often have focal amplifications that inflate IGH B cell fractions, and we aim to be conservative when calling somatic CNAs.

Because our method for calling germline CNVs was invalid for samples with high B cell fractions, we developed an adapted version for these cases. It followed a similar procedure but used only the final 20 kb of the *IGH* locus as a coverage baseline to avoid the effects of V(D)J recombination. We first divided the *IGH* locus into regions representing IGHV segments or class-switched segments. Next, we calculated mean coverage in 1kb windows, using the principle that coverage increases along the IGHV region due to fewer V segments being deleted in V(D)J recombination toward the locus's end. We performed this analysis in both directions to identify potential breakpoints inconsistent with V(D)J recombination. For example, a sudden drop and subsequent rise in coverage along the V segment region is unlikely due to V(D)J recombination but may indicate germline CNVs like copy number deletions. We then categorized these segments based on the change in coverage before and after the breakpoint, using the same method as the standard germline CNV caller.

Supplementary Fig. 2a gives an overview of the procedure for *IGH* germline haplotype and somatic CNA normalisation.

Additional validation of ImmuneLENS

IGH germline and somatic copy number validation

One complication of the *IGH* locus is the prevalence of germline copy number variants involving deletions and duplications[4]. These variants have been recently studied regarding disease susceptibility. However, when estimating B cell fractions, a large germline deletion in the *IGH* locus can be misinterpreted by the ImmuneLENS model as a V(D)J deletion event. To assess the scale of this issue, we applied the standard ImmuneLENS model to blood germline samples from the 100KGP pan-cancer and rare disease cohort. In previous studies, B cells comprise approximately 10% of circulating lymphocytes in healthy adults[5]. However, our calculations showed a substantial proportion with enriched (>10%) or high (>20%) B cell content as a percentage of all nucleated cells (Supplementary Fig. 2b). We hypothesized that these high B cell fractions were due to prevalent germline copy number variants. To test this, we examined all Father-Mother-Child trios within the 100KGP rare disease cohort. We categorized the parents' samples as normal, enriched (>10%), or high (>20%) and then examined the distribution of the child's B cell fraction (Supplementary Fig. 2c). We observed a clear inheritance pattern of high B cell fractions, suggesting they are due to germline copy number variants. By examining individual family trios, we identified clear cases of large germline variants affecting B cell fraction calculations. Supplementary Fig. 2d shows a case where both the mother and child have a ~242 kb deletion of one allele. This region lies within the IGHV locus (~106,082–106,324 kb) and contains 19 IGHV genes used in the ImmuneLENS model. It results in both the mother and child having a calculated B cell fraction of 0.35 (Supplementary Fig. 2d, right panels).

While the ImmuneLENS model for T cell fraction assumes somatic copy number alterations affecting TCR loci in tumour samples are unlikely, this is not the case for the *IGH* locus. Within

the TRACERx100 samples, we identified tumour samples with copy number changes in the *IGH* locus that are inconsistent with V(D)J recombination, such as tumor region 4 of CRUK0062 (Supplementary Fig. 2e). These alterations led to incorrect *IGH* B cell fraction calculations.

To correct for germline and somatic copy number alterations in the *IGH* locus before calculating the estimated IGH B cell fraction, we used our developed *IGH* copy number callers (see Supplementary Fig. 2a). Applying this to the 100KGP germline blood samples (Supplementary Fig. 2f) reduced the number of samples with enriched or very high B cell content. The previously highlighted mother and child B cell fractions, as well as the CRUK0062 R4 sample (Supplementary Fig. 2h), were reduced to a normal physiological range. Therefore, germline haplotype variants within the IGH locus likely explain the majority of our enriched and high IGH B cell fraction samples. An exception is one 100KGP pan-cancer participant identified with extremely high predicted circulating B cell content before correction (circulating *IGH* B cell fraction = 0.95, Supplementary Fig. 2i). However, hospital records show this participant had B-cell chronic lymphocytic leukemia (B-CLL) concurrently with their solid tumor, and the germline blood sample likely consisted almost entirely of these B cells.

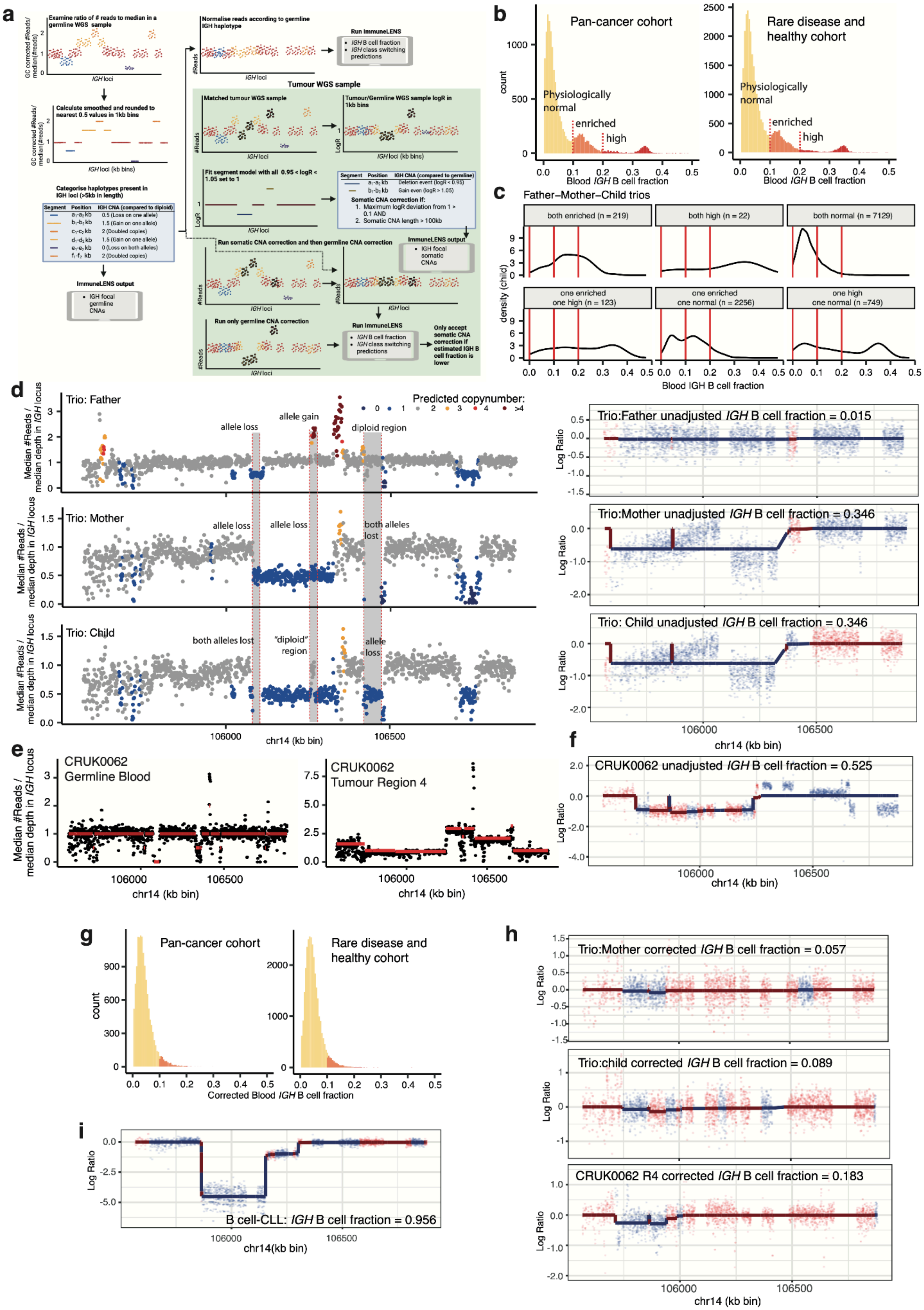

a
Examine ratio of # reads to median in a germline WGS sample
Calculate smoothed and rounded to nearest 0.5 values in 1kb bins
Categorise haplotypes present in IGH loci (>5kb in length)
ImmuneLENS output
IGH focal germline CNAs
Normalise reads according to germline IGH haplotype
Run ImmuneLENS
IGH B cell fraction
IGH class switching predictions
Tumour WGS sample
Matched tumour WGS sample
Tumour/Germline WGS sample logR in 1kb bins
Fit segment model with all 0.95 < logR < 1.05 set to 1
Somatic CNA correction if:
1. Maximum logR deviation from 1 > 0.1 AND
2. Somatic CNA length > 100kb
Run somatic CNA correction and then germline CNA correction
ImmuneLENS output
IGH focal somatic CNAs
Run only germline CNA correction
Only accept somatic CNA correction if estimated IGH B cell fraction is lower
b
Pan-cancer cohort
Rare disease and healthy cohort
Physiologically normal
enriched
high
count
Blood IGH B cell fraction
c
Father–Mother–Child trios
both enriched (n = 219)
both high (n = 22)
both normal (n = 7129)
one enriched one high (n = 123)
one enriched one normal (n = 2256)
one high one normal (n =749)
density (child)
Blood IGH B cell fraction
d
Trio: Father
Trio: Mother
Trio: Child
Predicted copynumber: 0 1 2 3 4 >4
allele loss
allele gain
diploid region
both alleles lost
"diploid" region
Median #Reads / median depth in IGH locus
chr14 (kb bin)
Trio:Father unadjusted IGH B cell fraction = 0.015
Trio:Mother unadjusted IGH B cell fraction = 0.346
Trio: Child unadjusted IGH B cell fraction = 0.346
Log Ratio
e
CRUK0062 Germline Blood
CRUK0062 Tumour Region 4
f
CRUK0062 unadjusted IGH B cell fraction = 0.525
g
Pan-cancer cohort
Rare disease and healthy cohort
Corrected Blood IGH B cell fraction
h
Trio:Mother corrected IGH B cell fraction = 0.057
Trio:child corrected IGH B cell fraction = 0.089
CRUK0062 R4 corrected IGH B cell fraction = 0.183
i
B cell-CLL: IGH B cell fraction = 0.956
chr14(kb bin)

Supplementary Figure 2

a. Cartoon overview of method to call germline *IGH* haplotype copy number alterations within the *IGH* loci and potential somatic CNA occurring in cancer cells. **b.** Histogram showing the distribution of circulating *IGH* B cell fraction in the 100KGP rare disease and healthy or pan-cancer cohort, separated by those samples with physiologically normal fractions (< 0.1), enriched (> 0.1 and < 0.2) and high fractions (>0.2). **c.** Density plots of circulating *IGH* B cell fraction for the child within a mother-father-child trio, grouped by the status of the parents calculated *IGH* B cell fraction into physiologically normal, enriched or high groups. **d.** Left panels: Normalised number of reads in 1kb bins within the *IGH* locus for a mother-father-child trio within the 100KGP. 1kb bins are coloured by the called copy number status in the ImmuneLENS methods, genomic regions showing clear inheritance patterns are highlighted. Right panels: Output of ImmuneLENS on non-corrected *IGH* coverage values. **e.** Normalised number of reads in 1kb bins for TRACERx patient CRUK0062, germline blood sample and tumour region 4. **f.** Output of ImmuneLENS on uncorrected coverage values of CRUK0062 tumour region 4. **g.** Histogram showing the distribution of circulating *IGH* B cell fraction in the 100KGP cohort using corrected coverage values for germline *IGH* haplotype variation called by ImmuneLENS. **h.** Output of ImmuneLENS on corrected *IGH* coverage values on the mother-father-child trio in C. and CRUK0062 tumour region 4. **i.** ImmuneLENS output of circulating *IGH* B cell fraction on uncorrected *IGH* coverage values for a participant that has B cell chronic lymphocytic leukaemia.

Comparison of ImmuneLENS and T cell ExTRECT on TRACERx WGS vs WES

We applied the ImmuneLENS model to matched TRACERx WES and WGS data, comparing the results to T cell ExTRECT values from WES data. The ImmuneLENS and T cell ExTRECT T cell fractions from WES data were highly correlated (ρ = 0.92, P= 0, Supplementary Fig. 3a). Similarly, ImmuneLENS values from WES and WGS data were highly correlated (ρ = 0.77, P = 2.27×10^{-72}, Supplementary Fig. 3b). However, compared to the Danaher T cell score[6] (Supplementary Fig. 3c), the ImmuneLENS method showed higher correlation on WES data and significantly higher on WGS data (T cell ExTRECT (WES) ρ = 0.6, P = 2.18×10^{-47}; ImmuneLENS (WES) ρ = 0.64, P = 1.43×10^{-60}; ImmuneLENS (WGS) ρ = 0.84, P = 0).

For samples with low T cell infiltration by T cell ExTRECT WES (<0.02), we found that ImmuneLENS again showed improved correlations with the Danaher T cell score (Supplementary Fig. 3c) (T cell EXTRECT (WES) ρ = 0.07, P = 0.36; ImmuneLENS (WES): ρ = 0.41 , P = 9.3×10^{-8}; ImmuneLENS (WGS): ρ = 0.71 , P = 1.05×10^{-6}). This suggests the WGS method is more sensitive than the exome approach for detecting low T cell fractions. However, we do not recommend using ImmuneLENS on WES data because the model does not account for probe biases in exome capture kits.

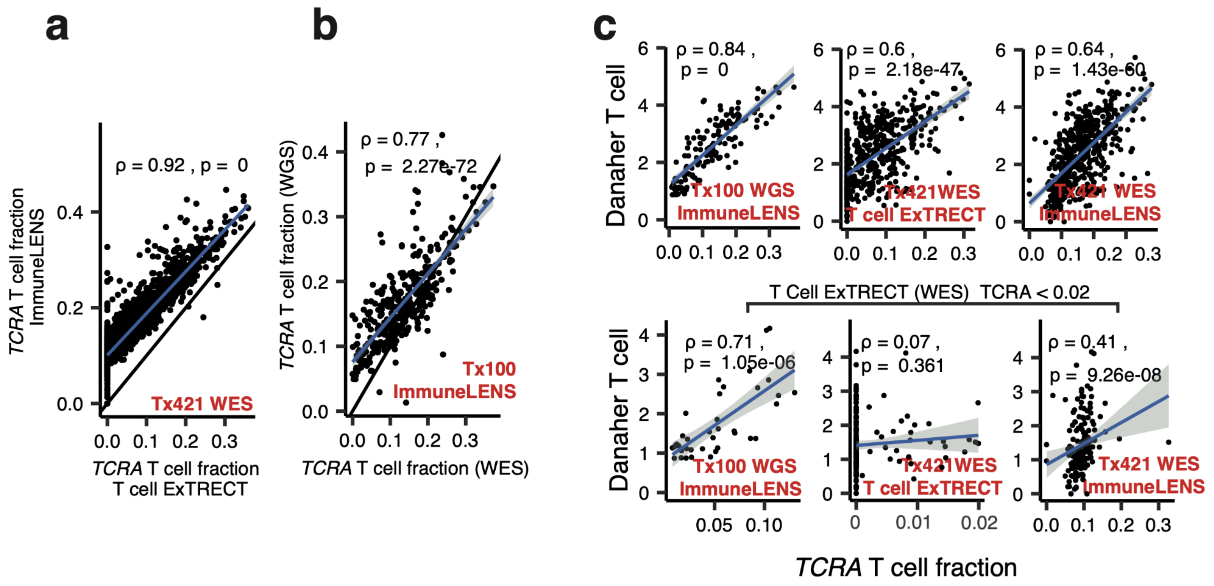

Supplementary Figure 3

a. Comparison of ImmuneLENS and T cell ExTRECT scores on the TRACERx421 WES data. Black line represents the y = x line. **b.** Comparison of ImmuneLENS on either the TRACERx100 WES and matched WGS data. **c.** Scatter plot of Danaher T cell scores versus *TCRA* T cell fraction as measured from either WGS or WES and either using the T cell ExTRECT method (for WES) or the ImmuneLENS method (for WES and WGS). Bottom panels: samples with < 0.02 estimated T cell fraction from T cell ExTRECT in WES. *IGH* germline copy number validation using 100KGP cohort. The blue lines represent the line of best fit and shaded grey region 95% confidence interval. P values for Spearman's ρ were derived from a two tailed t-distribution using the correlation coefficient and sample size.

RNAseq validation of *IGH* class switching in the TRACERx100 cohort and scRNA data

As an orthogonal method, we correlated IgA, IgG, and IgM/D proportions with those calculated using the TRUST[7] method from RNAseq data (Supplementary Fig. 3a-c). We also compared these proportions to the number of unique IGHV clones identified by MiXCR on the RNAseq data (Supplementary Fig. 3d-f). We identified a significant correlation only with the number of IGHV clones from MiXCR[8] (Supplementary Fig. 3d: ρ = 0.48, P = 3 x 10^{-12}). There was no significant correlation with TRUST IgG calls (Supplementary Fig. 3c: ρ = 0.051, P = 0.5) or with IgA calls from either TRUST or MiXCR.

We hypothesise that the lack of concordance between the IgA B cell fraction and RNAseq data is partly due to biological differences in gene expression levels among different class-switched B cells. To test this, we examined a non-small cell lung cancer scRNA dataset[9] and categorized B and plasma cells into IgA, IgG, or IgM/D based on IGH class-switch gene expression. After removing B cells with unclear class-switch status, we found that the majority of plasma cells

were enriched for IgG B cells (Supplementary Fig. 3g). We also observed that plasma cells had higher read counts than B cells (Supplementary Fig. 3h), which, together with our data, suggests that IgG plasma cells may dominate signals from RNAseq data.

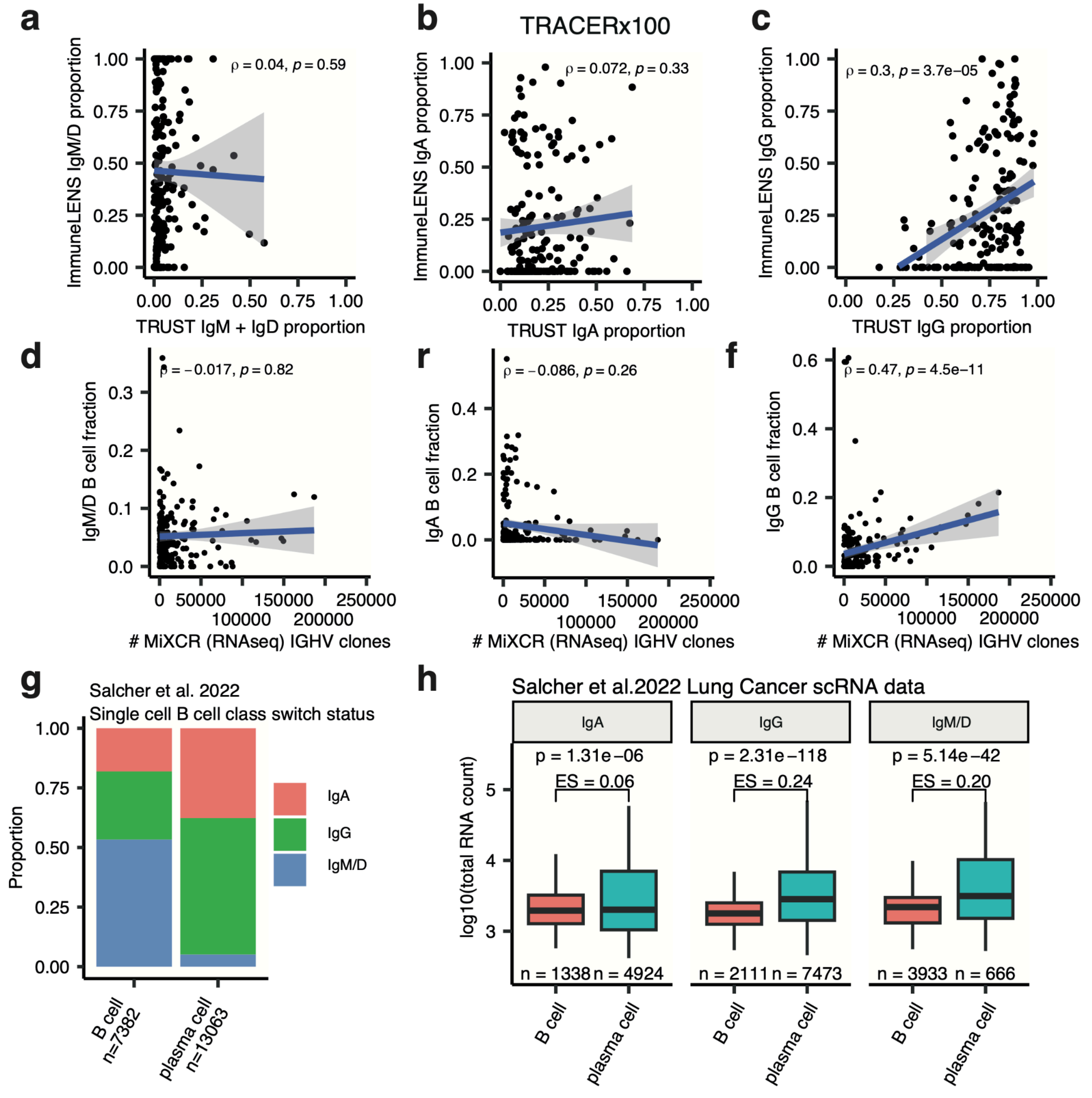

Supplementary Figure 4:
a-c. Correlation of proportion of RNA reads mapping to *IGH* from the TRUST algorithm with different class switched proportions to the proportion of class switched B cells as measured from DNA with the ImmuneLENS method in TRACERx100. **d-f.** Correlation of the number of unique IGHV clones as measured by MiXCR from RNAseq data to sample matched B cell fraction as measured by WGS for either IgM/D, IgG or IgA B cells. **g.** Class switched status of B cell subsets in scRNA Salcher et al. data. **h.** Number of reads by class switch status of different B cell subsets, with significance tested with

two-sided Wilcoxon rank-sum tests. In **a-f** the blue line represents the line of best fit and the shaded grey region the 95% confidence interval.Boxplots in **h** show the median, lower and upper quartile and with whiskers extending to 1.5 times the interquartile range above and below the interquartile range. P values for Spearman's ρ were derived from a two tailed t-distribution using the correlation coefficient and sample size.

IGH B cell fraction validation using the 1000 genomes cohort

To further validate our B cell fraction and class-switching predictions, we downloaded 2,557 high-depth WGS files (average depth = 34X) from the 1000 Genomes cohort[10]. These samples are derived from lymphoblastoid cell lines (LCLs), which are Epstein-Barr virus-transformed B lymphocytes typically sourced from blood samples. As expected, these samples had an extremely high proportion of B cells (Supplementary Fig. 5a). Although LCLs in culture are expected to become clonal within 8 weeks[11], we detected samples with high diversity of B cell clonotypes and various IGHV segments used in the fitted models (Supplementary Fig. 5b). The observed B cell fraction was often less than one, with some samples near 0.5. This is likely due to allelic exclusion at the *IGH* locus, where only one allele undergoes V(D)J recombination[12]. Therefore, our B cell fractions underestimate the true values because they cannot account for B cell clonotypes with an un-recombined, non-functional *IGH* locus due to allelic exclusion. Assuming all our LCL samples are 100% B cells, we can estimate the percentage undergoing allelic exclusion. In the most polyclonal LCL samples (IGHV Shannon diversity >2), we predict that a median of 35% of B cells have undergone allelic exclusion (Supplementary Fig. 5c-d). From the entire 1000 Genomes cohort, we identified samples that were highly polyclonal or completely clonal, with differing proportions of class-switched B cells (Supplementary Fig. 5e).

To validate our class-switching predictions, we obtained processed transcriptomic data from the GEUVADIS study[13] and conducted a differential gene expression analysis between predicted B cell class-switching groups. Despite the polyclonal nature of many samples and the possibility that DNA and RNA samples taken at different time points may not share the same polyclonal structure, we identified numerous significant genes after multiple hypothesis adjustment. Specifically, we found genes up- and downregulated for IgM/D (808 up, 615 down), IgG (536 up, 587 down), and IgA (32 up, 40 down). In particular, *IGHM* and *IGHD* were significantly overexpressed in samples with high numbers of non-class-switched B cells measured from DNA (*IGHM*: logFC = 2.98 , adj p =1.4×10^{-32} , *IGHD*: logFC = 2.35, adj p = 1.1×10^{-28}). Similarly, the IGHG genes were all significantly upregulated in the IgG comparison (*IGHG1:* logFC = 2.55 , adj p =5.9×10^{-21}, *IGHG2:* logFC = 1.61 , adj p =5.1×10^{-11}, *IGHG3:* logFC = 1.64 , adj p =1.6×10^{-10}, *IGHG4:* logFC = 2.15 , adj p =7.9×10^{-23}). The IGHA genes were also significantly upregulated in the IgA comparison (*IGHA1:* logFC = 2.67 , adj p =4.2×10^{-16}, *IGHA2:* logFC = 2.15 , adj p =8.3×10^{-14}) (Supplementary Fig. 5f). We note that significant transcriptomic changes between class-switched B cells (1,423 genes for IgM/D, 1,123 genes for IgG) may confound eQTL studies based on data from LCL cultures. We have released our full classification of all 1000 Genomes samples as a resource to enable control in future analyses or selection for studies involving different types of class-switched LCLs (see Data Availability).

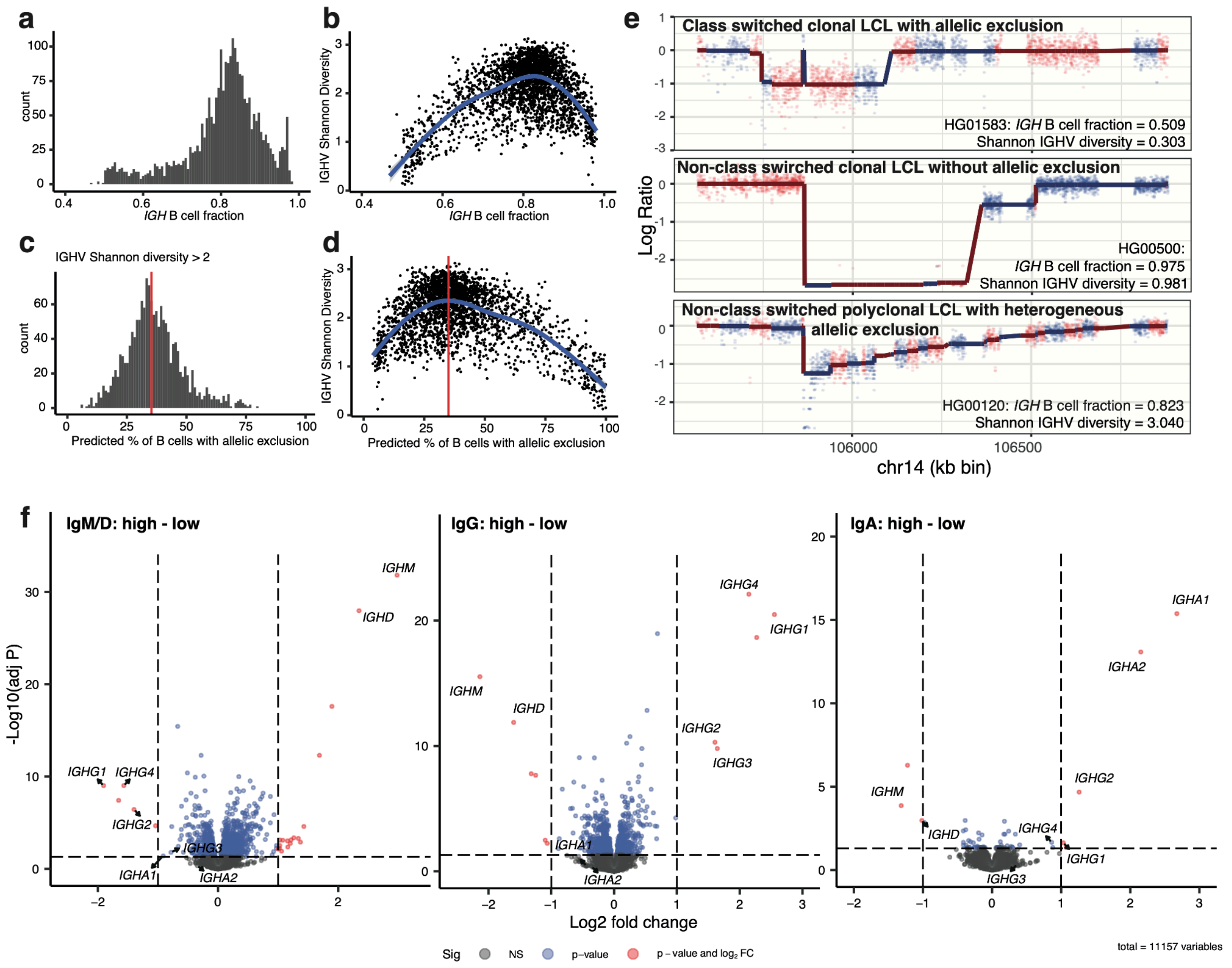

Supplementary Figure 5

a. Histogram distribution of *IGH* B cell fraction in the 1000 genome cohort. **b.** Scatter plot of IGHV Shannon diversity vs *IGH* B cell fraction with the blue line representing the fitted smooth line from a loess model. **c.** Histogram of percent of B cells predicted to have undergone allelic exclusion for most diverse samples from the 1000 genomes cohort (IGHV shannon diversity > 2), red line represents the median value 30% **d.** Scatter plot of the percent of B cells predicted to have undergone allelic exclusion versus the IGHV Shannon diversity, blue fitted line is from a loess model and red line represents the value of 35% B cells underground allelic exclusion. **e.** ImmuneLENS output for *IGH* B cell fraction of three samples within the 1000 genome LCL cohort. **f.** Volcano plots of limma voom analysis accounting for multiple hypothesis testing of the Geuvadis 1000 genome RNAseq data with samples separated into high and low by the median of the IgM/D, IgG or IgA B cell fractions. Dashed lines represent cut-offs for -log10(adj p) at -log10(0.05) and Log2 fold change > 1 or < -1.

Performance of ImmuneLENS on matched high and low depth WGS data sets

We validated these results using datasets with matched low- and high-depth WGS data. We first utilised low-depth TCGA samples (median depth = 4.95X) matched with high-depth samples from the PCAWG study (blood median depth = 37.3X, tumor median depth = 51.2X). We identified significant correlations with both circulating and infiltrating T cell fractions (Supplementary Fig. 6a) (*TCRA*: Circulating: ρ = 0.86, P = 0, Infiltrating: ρ = 0.64, p =1.17×10^{-16}; *TCRB*: Circulating: ρ = 0.72, p = 0, Infiltrating: ρ = 048, p = 5.9×10^{-9}; *TCRG:* Circulating: ρ = 0.81, p = 3.14×10^{-29}, Infiltrating: ρ = 0.56, p = 3.21×10^{-12}).

To ascertain the accuracy of *TCRA* T cell fractions from low-depth WGS at low levels, we filtered the high-coverage PCAWG samples. We selected samples below varying thresholds (0.2, 0.15, 0.1, and 0.05; Supplementary Fig. 6b). For samples below 0.2, the accuracy remained high (ρ = 0.73, p = 0), as it did for samples below 0.15 (ρ = 0.64, p = 1.2×10^{-6}). In both cases, the concordance—the percentage of low-coverage samples also below 0.2 and 0.15, respectively—was high (88.1 and 88%). For samples below 0.1, the correlation was no longer significant (ρ = 0.26, p = 0.18), though the concordance remained high (86.2%), indicating they were still calculated as low fractions in the low-depth samples. For samples below 0.05 in the high-depth data, there was neither a significant correlation (ρ = 0.31, P = 0.25) nor high concordance; only 40% were also below 0.05 in the low-depth samples.

For *IGH* B cell fraction, we observed a much higher correlation without copy number corrections (Supplementary Fig. 6c) (Circulating: ρ = 0.66, p = 5.52×10^{-17}, Infiltrating: ρ = 0.6, p = = 3.32×10^{-14}), compared to with corrections (Circulating: ρ = 0.38, p = 0.00088, Infiltrating: ρ = 0.29, p = 0.0016). This suggests that higher coverage is necessary for accurate *IGH* locus copy number calling.

We used low-depth 1000 Genomes samples of lymphoblastoid cell lines (LCLs, median depth = 1.25X) to assess the accuracy of class-switching predictions. We noticed that many low-depth samples had a very low estimated B cell fraction but a high *TCRA* T cell fraction score (Supplementary Fig. 6d). We assumed these were newly established LCLs not yet dominated by B cells and still containing substantial fractions of other cell types. Therefore, we removed any sample with an IGH B cell fraction below 0.5 from our analysis. Following this, we identified significant correlations between high- and low-depth samples for *IGH* B cell fraction (ρ = 0.65, p = 1.21×10^{-182}), IgA (ρ = 0.61, p = 1.38×10^{-309}), IgG (ρ = 0.81, p = 0) and IgM/D B cell fraction (ρ = 0.65, p = 0) (Supplementary Fig. 6e).

Using the low-depth WGS samples (excluding those with low total *IGH* B cell fraction), we classified samples into high and low class-switched groups and replicated the LIMMA analysis presented in Supplementary Fig. 5f. Supplementary Fig. 6f shows that using the low-coverage WGS samples, we still identified significant upregulation of relevant class-switched B cell gene segments. Comparing the calculated log fold change values from the LIMMA analysis using either high or low coverage, we observed notable concordance (Supplementary Fig. 6g).

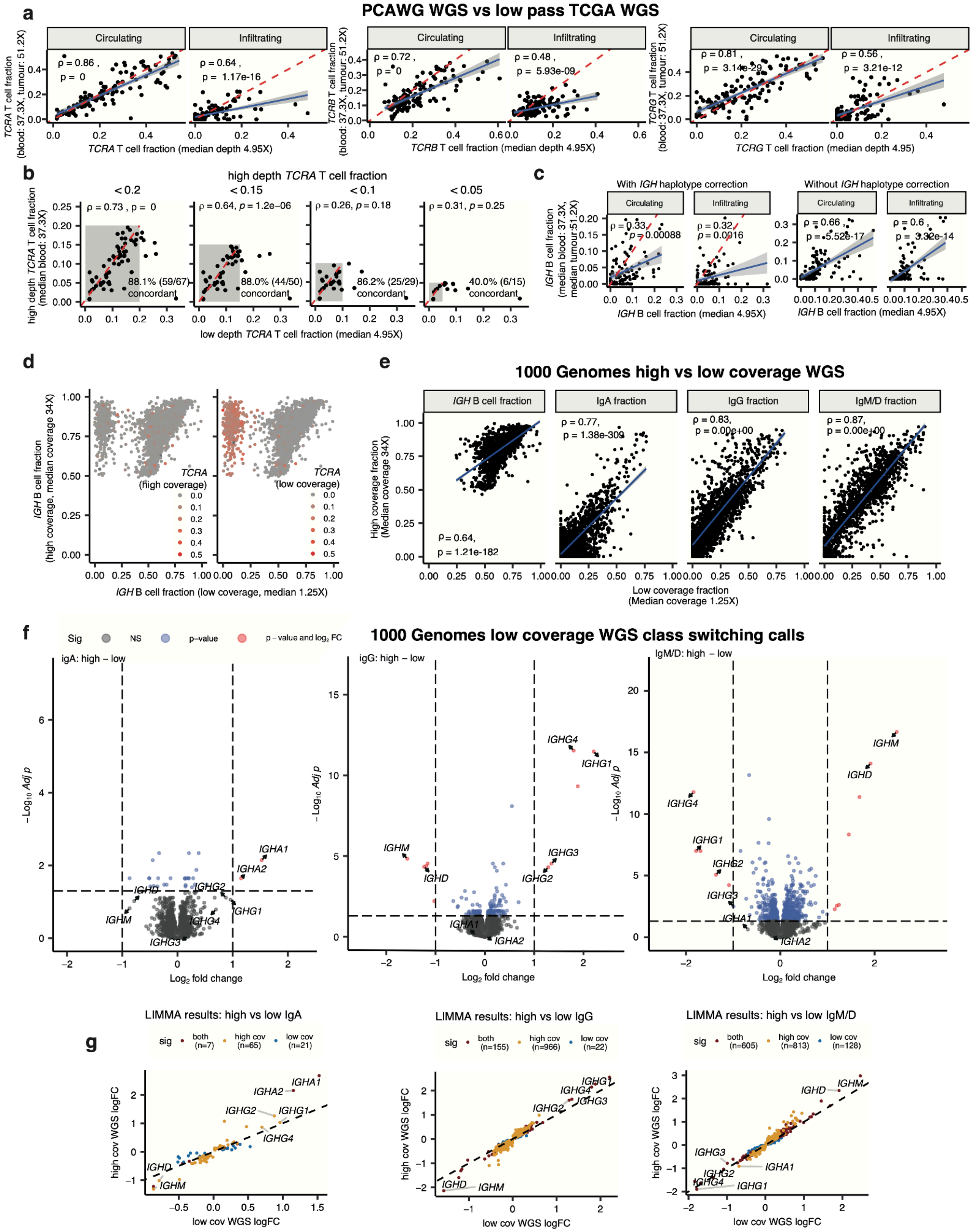

Supplementary Figure 6

a. Scatter plots for T cell fraction as measured by *TCRA, TCRB* or *TCRG* on the matched high coverage WGS samples (median coverage blood samples 37.3X, tumour samples 51.2X) with low pass WGS

TCGA data (median coverage 4.95X). **b.** Scatter plots for circulating *TCRA* T cell fraction between high and low coverage WGS filtered by the high depth fraction being lower than varying thresholds (0.2, 0.15, 0.1 and 0.05) signified by shaded grey regions, dotted black line represents the line y = x **c.** Scatter plots for IGH B cell fraction calculated with or without haplotype correction on the high coverage PCAWG data versus lowpass TCGA. **d.** *IGH* B cell fraction calculation on the high coverage 1000 genome data (median coverage 34X) versus the matched low coverage data (1.25X), points are coloured by calculated *TCRA* T cell fraction from the high coverage data (left panel) and the low coverage data (right panel). **e.** Scatter plots for *IGH* class switching fractions for high versus low WGS data with samples with < 0.5 *IGH* B cell fraction in the low coverage cohort removed. **f.** Volcano plots showing differentially regulated genes as calculated by LIMMA. High and low class switched fraction groups in the 1000 genomes cohort were calculated from the low coverage WGS data, **g.** Comparison of significant genes from limma-voom analysis, accounting for multiple hypothesis testing, in the 1000 genomes cohort using either the low or high coverage WGS data to calculate high and low class switched fraction groups. Dashed lines represent cut-offs for -log10(adj P) at -log10(0.05) and Log2 fold change > 1 or < -1. In **a, c** and **e** the blue lines represent the line of best fit and grey regions the 95% confidence interval. P values for Spearman's ρ in were derived from a two tailed t-distribution using the correlation coefficient and sample size.

Use of ImmuneLENS in additional pan-cancer WGS data sets

Our results on the pan-cancer landscape of circulating T and B cell fractions rely primarily on the 100KGP dataset, which uses a consistent DNA sequencing protocol and detailed matched clinical data. Other datasets using WGS blood samples may have differing protocols, particularly in the timing and method of DNA sequencing, which could affect T and B cell quantification using ImmuneLENS. To investigate these effects, we applied ImmuneLENS to a subset of the PCAWG/TCGA WGS dataset with matched blood and tumor samples (n = 539) (Supplementary Fig. 7a). We also examined the TCGA data, using the WES version of T cell ExTRECT to calculate lymphocyte scores in 6,189 tumour and 5,492 blood samples (Supplementary Fig. 7b).

We observed many of the same trends in this cohort as in the 100KGP, such as low levels of circulating T cell fractions in glioblastoma. However, we could not confirm the timing of germline blood samples relative to surgery or treatments for these cases. For the PCAWG data, we noted many cases of extremely high germline blood *TCRA* fractions, especially within the colorectal cohort (Supplementary Fig. 7c). Many values were above the 95th and 99th percentiles of circulating *TCRA* T cell fractions (0.28 and 0.37) in the 100KGP cohort. This could be related to the TCGA treatment center that sequenced the samples, but we consider a treatment-related effect more likely. One notable colorectal cancer case had an estimated *TCRA* T cell fraction of ~0.5 and, when examined, revealed a clear signal of V(D)J recombination with a high TRAV Shannon diversity (Supplementary Fig. 7d).

Taking all germline blood samples within the PCAWG data, we saw no significant differences between males and females (Supplementary Fig. 7e). However, when we include only samples with *TCRA* T cell fractions below the 95th percentile of the 100KGP quantile, a significant association emerges. In contrast, within the TCGA WES data, we detected a significant difference between male and female fractions (Supplementary Fig. 7e). This leads us to suspect

an unaccounted factor is causing increased T cell fractions in a minority of the PCAWG germline samples. In the absence of comprehensive clinical information on these germline samples, especially their timing related to treatment, it is extremely difficult to explain these differences. This highlights an important issue when using ImmuneLENS with WGS germline samples. In many datasets, these samples were collected not as measurements of time-dependent variables but as presumed unchanging germline controls for cancer analysis.

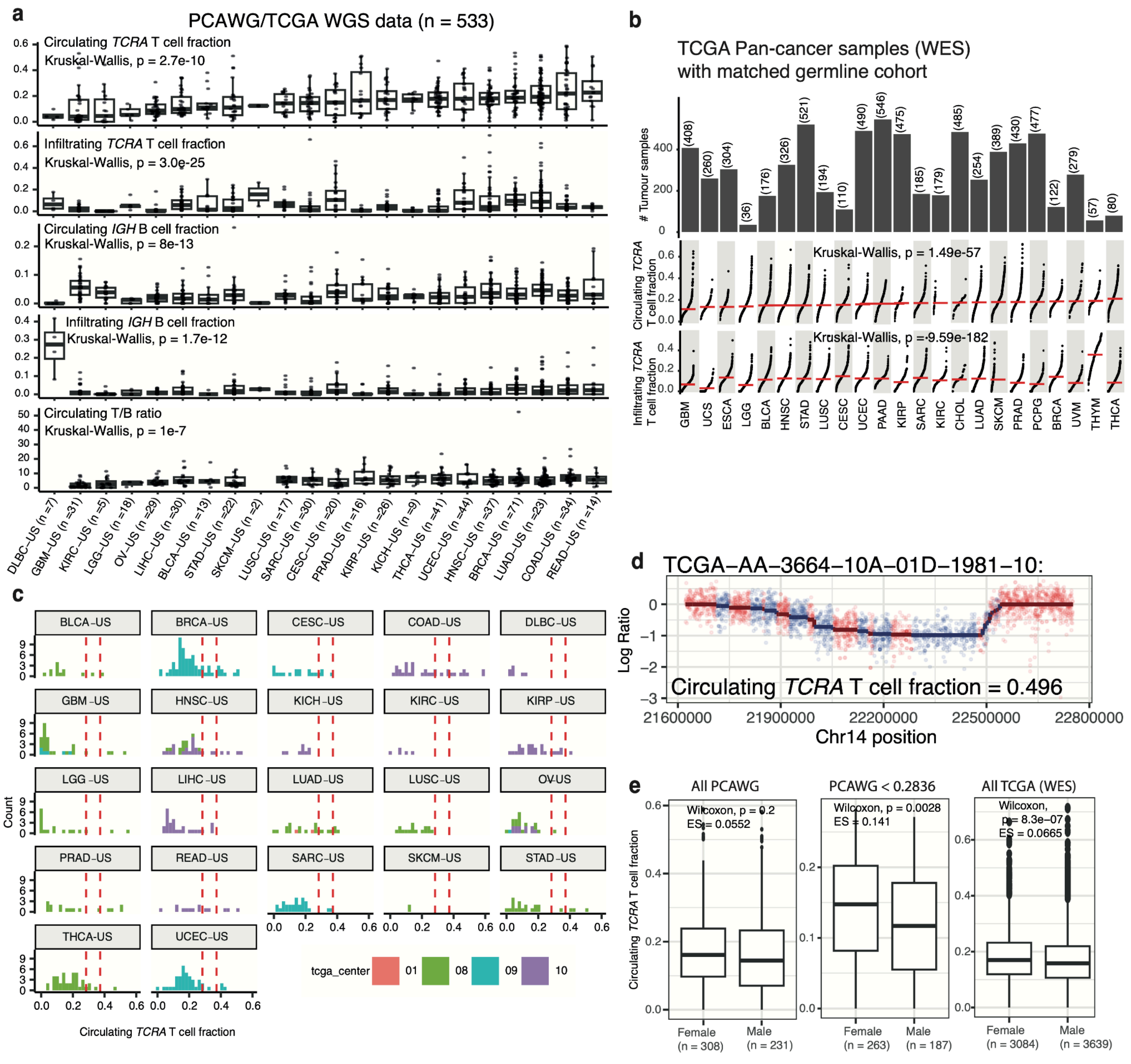

Supplemental Figure 7

a. Overview plot of output of ImmuneLENS run in the subset of the PCAWG data set composed of TCGA samples. **b.** Overview plot of T Cell ExTRECT run on the TCGA WES data. **c.** Circulating *TCRA* T cell fraction within the PCAWG cohort coloured by TCGA sequencing centre. Dashed red lines represent the 95 and 99 percentiles of the 100KGP pan-cancer circulating *TCRA* T cell fractions. **d.** Example output of case within the PCAWG data with very high circulating *TCRA* T cell fraction. **e.** Boxplots showing differences between males and females within PCAWG and TCGA, with 0.2836 representing the 95%

percentile of *TCRA* T cell fraction scores calculated in the 100KGP data set with significance tested with a two sided Wilcoxon rank-sum test. Boxplots in **a** and **e** show the median, lower and upper quartile and with whiskers extending to 1.5 times the interquartile range above and below the interquartile range.

Extended analysis of 100KGP

ImmuneLENS reveals differences in TCR repertoire

We investigated whether ImmuneLENS could uncover differences in TRAV segment usage between tumour and blood samples and among cancer histologies. We restricted analysis to samples with *TCRA* T cell fraction > 0.05 and used propensity matching to create cohorts with similar T cell fraction distributions to test for differences in TRAV segment usage. Between blood and tumour samples, we found 29 TRAV segments with significant usage differences (Supplemental Data, Supplementary Fig. 8a), signifying distinct circulating and infiltrating T cell repertoires. We then tested for significant differences in the infiltrating T cell repertoire among cancer histologies. Significant usage differences were identified in B cell-derived hematological cancers (Supplementary Fig. 8a), with a substantial increase in TRAV41 usage (58% vs. 23% in the propensity-matched cohort, p = 0.0001). This was driven almost entirely by acute lymphoblastic leukemia (*TRAV41* usage 81% vs 30% in propensity matched cohort, P = 4.3x 10^{-6} Supplementary Fig. 8a). Only a minority of ALL cases in the 100KGP cohort were T-cell-derived ALL (7.9%, 24/304), with the majority being either of unknown origin due to lack of clinical data (40%, 124/304) or B cell ALL (51%, 156/304). While aberrant V(D)J recombination involving *TRAV41* has been reported in T-ALL[14], we suspect the predicted TRAV41 usage may actually be the downstream TRDV2 segment not included in the standard TCRA ImmuneLENS model. *TCRD* rearrangements involving the *TRDV2* segment have been shown to be common within B-ALL[15].

Another histology group with significant alterations in TRAV segment usage was mucosal gastrointestinal cancer types (colon adenocarcinoma, rectum adenocarcinoma, colorectal and upper GI neuroendocrine, stomach adenocarcinoma, and other colorectal cancers) (Supplementary Fig. 8a). This group was characterised by increased *TRAV1-2* usage (17% vs 11%, p = 7.5 10^{-4}) and decreased *TRAV20* usage (20% vs 31%, p = 1.1 10^{-6}) compared to a propensity matched cohort. We hypothesise that this is a signal of mucosal associated invariant T (MAIT) cells which exclusively use the *TRAV1-2* segment.

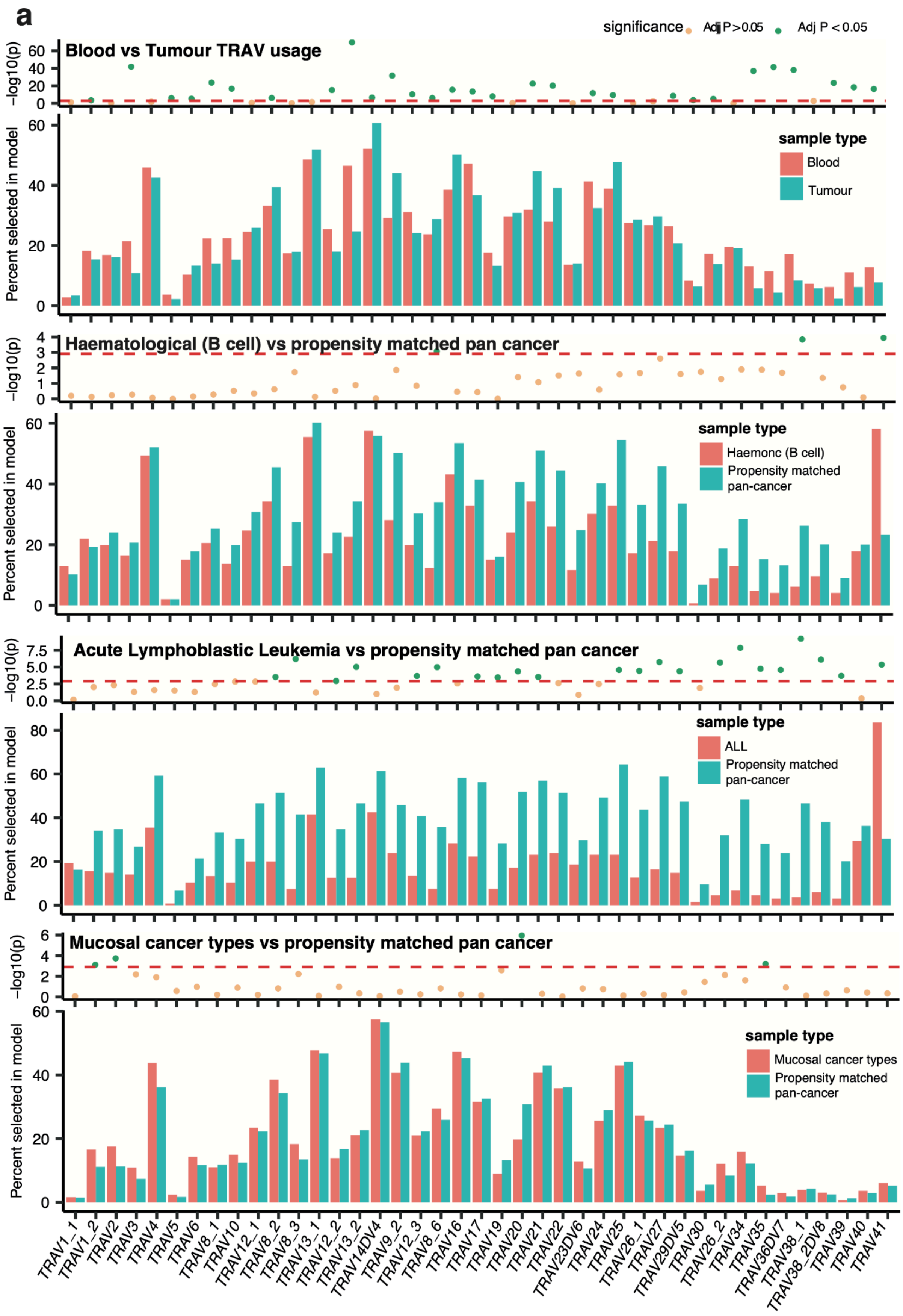

a
significance
Adj P > 0.05
Adj P < 0.05
Blood vs Tumour TRAV usage
−log10(p)
Percent selected in model
sample type
Blood
Tumour
Haematological (B cell) vs propensity matched pan cancer
Haemonc (B cell)
Propensity matched pan-cancer
Acute Lymphoblastic Leukemia vs propensity matched pan cancer
ALL
Mucosal cancer types vs propensity matched pan cancer
Mucosal cancer types
TRAV1_1
TRAV1_2
TRAV2
TRAV3
TRAV4
TRAV5
TRAV6
TRAV8_1
TRAV10
TRAV12_1
TRAV8_2
TRAV8_3
TRAV13_1
TRAV12_2
TRAV13_2
TRAV14DV4
TRAV9_2
TRAV12_3
TRAV8_6
TRAV16
TRAV17
TRAV19
TRAV20
TRAV21
TRAV22
TRAV23DV6
TRAV24
TRAV25
TRAV26_1
TRAV27
TRAV29DV5
TRAV30
TRAV26_2
TRAV34
TRAV35
TRAV36DV7
TRAV38_1
TRAV38_2DV8
TRAV39
TRAV40
TRAV41

Supplementary Figure 8

a. Percentage of samples with TRAV segments at fractions > 0.001 in different cohorts compared to propensity matched for *TCRA* T cell fraction cohorts of the same size with P values (top panel) calculated using Chi-squared tests to identify segments used at significantly different proportions.

Fold change analysis of circulating lymphocyte fractions between males and females in the healthy and cancer cohort

We quantified the fold change (FC) between females and males for lymphocyte counts using a bootstrap method to obtain 95% confidence intervals (see Supplementary Data). Across the pan-cancer cohort, females had a 21% higher circulating *TCRA* T cell fraction than males ($p = 1.3 \times 10^{-70}$). The *IGH* B cell fraction was 6.9% higher in females ($p = 4.6 \times 10^{-8}$), driven entirely by the IgM/D subpopulation (15% higher, $p = 1.9 \times 10^{-10}$), while the class-switched B cells showed no significant difference. In contrast, in the healthy cohort, females had decreased total *IGH* B cells (3% decrease, $p = 3.4 \times 10^{-6}$) and IgM/D B cells (7.3% decrease, $p = 6.5 \times 10^{-14}$) compared to males. There was only a small increase in *TCRA* T cell fraction in females (1.2% increase, $p = 0.0017$) (Supplementary Fig. 9). The T/B cell ratio was also significantly higher in females than males (pan-cancer: 11% higher, $p = 2.4 \times 10^{-12}$), suggesting that sex-related differences in immune infiltrate do not solely reflect neutrophil levels.

We observed higher circulating *TCRA* T cell fractions in females compared to males ($FC > 1$ with $p < 0.05$) across 12 different cancer histologies (Supplementary Fig. 9). This remained significant after multiple hypothesis testing in the following cancers: lung squamous carcinoma (FC= 1.34, adj $p = 2.0 \times 10^{-5}$), sarcoma (FC= 1.19, adj $p = 2.0 \times 10^{-4}$), lung adenocarcinoma (FC= 1.16, adj $p = 4.5 \times 10^{-2}$) and colon adenocarcinoma (FC= 1.14, adjusted $P= 2.0 \times 10^{-5}$). In contrast, limited sexual dimorphism was observed in infiltrating lymphocytes. Overall IgM/D levels in the pan-cancer cohort were reduced in females (FC = 0.88, adjusted $p = 0.15$). Additionally, renal cell carcinoma showed higher *TCRA* T cell fractions in males than females (28.7% higher, adjusted $p = 0.0016$), consistent with previous work[16]. These data suggest that biological sex significantly impacts circulating lymphocyte levels in the blood of cancer patients. However, associations between sex and lymphocyte infiltrate in tumors are restricted to lung adenocarcinomas and renal cancers. Only weak associations are observed with the circulating T cell content of healthy individuals.

Next, we examined the differences in circulating lymphocyte fractions between healthy individuals and cancer patients. Using propensity matching, we selected cohorts with the same age and sex distribution for each disease type. We found that T cell fraction, *IGH* B cell fraction, and IgM/D B cells were significantly decreased in cancer patients compared to healthy controls (*TCRA*: 18% decrease, $p = 5.1 \times 10^{-342}$, *IGH*: 5.3% decrease, $p = 2.2 \times 10^{-10}$, IgM/D: 21% decrease, $p = 1.6 \times 10^{-85}$). In contrast, class-switched B cells, both in fraction and as a proportion of total B cells, were increased (Supplementary Fig. 9, right panels). Notably, the T/B cell ratio was also significantly decreased by 17% in cancer patients compared to healthy

participants (p = 3.7 x 10^{-106}). Thus, these differences cannot be solely explained by altered neutrophil levels.

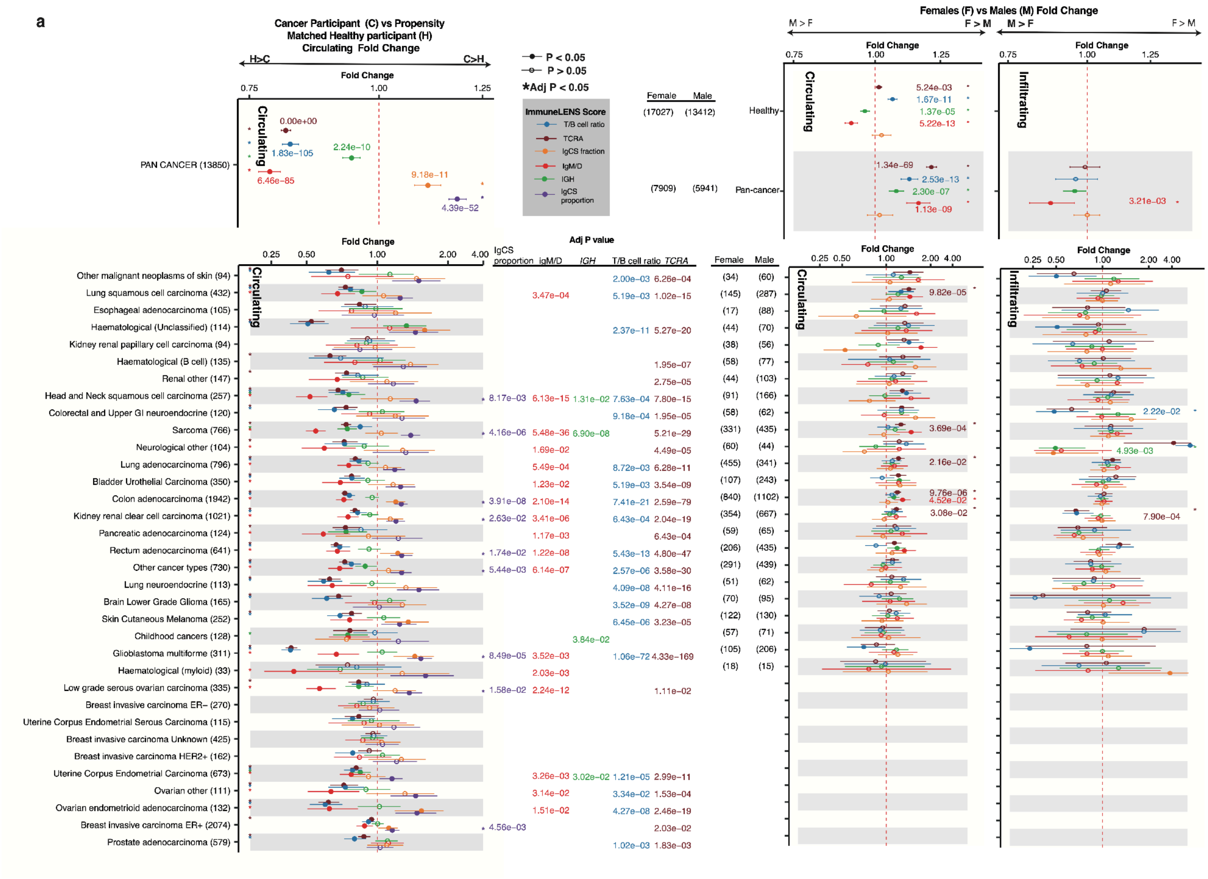

Supplementary Figure 9

A. Left panels: ratio of circulating ImmuneLENS fraction from cancer patients with propensity matched for age and sex 100KGP participants within the healthy cohort. Dotted red line is at fold change = 1. Right panels: fold change and 95% confidence interval of female versus males for ImmuneLENS fraction for both circulating and tumour infiltrating fractions. P values obtained from a bootstrapping method with 1000 bootstrap replicates.

GWAS SNPs associated with circulating *TCRA* T and *IGH* B cell fraction

Due to the smaller cohort size, our power to identify significant SNPs in the pan-cancer cohort is reduced. Despite this, some SNPs highly significant in the healthy European ancestry cohort showed no trend toward significance in the pan-cancer cohort. Two examples are rs2853946 within the *HLA-B* locus, linked to white blood cell count, and rs2919917, an intron variant in IL7

linked to lymphocyte counts. Both SNPs were highly significant in the European ancestry healthy cohort (-logp = 6.60 and 7.12, respectively, from PLINK) but were non-significant in the pan-cancer cohort (Supplementary Fig. 10a). The only significant SNP in the pan-cancer cohort was rs7919533, a variant within the CDH23 gene previously associated with monocyte counts (-logp = 4.84, Figure 3E). This analysis for the pan-cancer cohort is likely underpowered. For instance, variant rs3785549 within the PSMD3 gene was highly significant in the healthy cohort (-logp = 13.5) but only near the significance threshold in the cancer cohort (-logp = 3.02, Supplementary Fig. 10b).

To determine whether germline SNPs were affecting T cells directly or neutrophil levels, we repeated the PLINK analysis examining the T/B cell ratio. In this case, we found only one significant association in the African ancestry healthy cohort, which was with the Duffy SNP but instead rs6058796 (-logp = 5.47). Implying that the effect from the Duffy SNP is, as expected, completely driven by neutrophil levels (Supplementary Fig. 10c). We identified seven significant SNPs in the European ancestry healthy cohort (Supplementary Data) but none in the pan-cancer cohort.

We additionally looked for associations between SNPs and the IGH B cell fraction (Supplementary Fig. 10d). None were significant in the pan-cancer setting, but two SNPs were significant in the European ancestry healthy cohort. When dividing by class-switching status, one of these hits, rs56356600, was also found to be associated with IgM/D B cell fraction (*IGH:* -logp = 5.2, IgM/D: -logp = 7.1,Supplemental Data, Supplementary Fig. 10e). rs56356600 is an intron variant within *EBF1*, a key B cell transcription factor. There was limited overlap with SNPs significant for *TCRA* T cell fraction, T/B cell ratio, and *IGH* B cell fraction, as shown in an upset plot (Supplementary Fig. 10f). Only two SNPs were significant in both the T/B cell ratio and *TCRA* analyses (rs113542380 and rs10774625).

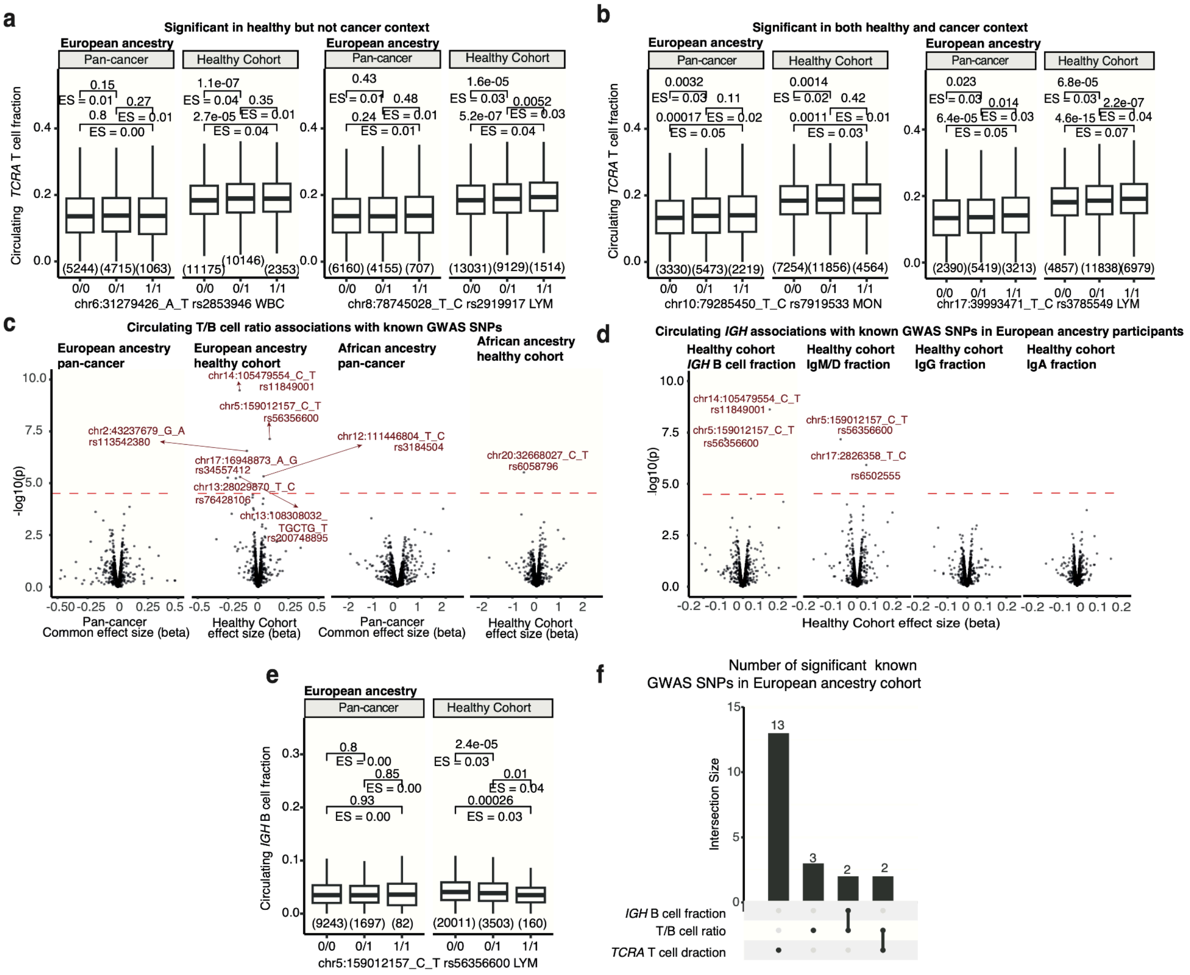

Supplementary Figure 10

a-b. Boxplots showing the effect size associated with the presence of different significant SNPs across the different ancestry cohorts. **c-d.** Volcano plots for tested known GWAS SNPs association with T/B cell ratio and *IGH* B cell fraction. **e.** Boxplots showing the effect size associated with the presence of different significant SNPs associated with *IGH* B cell fraction in the European ancestry cohorts **f.** Intersection of significant SNPs from known GWAS studies associated with circulating *TCRA* T cell fraction, *IGH* B cell fraction and T/B cell ratio. Boxplots in **a, b** and **e** show the median, lower and upper quartile and with whiskers extending to 1.5 times the interquartile range above and below the interquartile range. P values in **a, b** and **e** from two sided Wilcoxon rank-sum tests. The p-values in **c** and **d** are derived from the PLINK software that uses a linear regression model and performs a Wald test for each SNP, for the cancer cohort this was done separately for each histology and the p-values were combined using a meta-analysis with a common effects model.

Supplementary References

1. Bentham, R. *et al.* Using DNA sequencing data to quantify T cell fraction and therapy response. *Nature* **597**, 555–560 (2021).
2. Van Loo, P. *et al.* Allele-specific copy number analysis of tumors. *Proceedings of the National Academy of Sciences* **107**, 16910–16915 (2010).
3. Kuri-Magaña, H. *et al.* Non-coding Class Switch Recombination-Related Transcription in Human Normal and Pathological Immune Responses. *Front. Immunol.* **9**, 2679 (2018).
4. Collins, A. M., Yaari, G., Shepherd, A. J., Lees, W. & Watson, C. T. Germline immunoglobulin genes: disease susceptibility genes hidden in plain sight? *Curr Opin Syst Biol* **24**, 100–108 (2020).
5. Morbach, H., Eichhorn, E. M., Liese, J. G. & Girschick, H. J. Reference values for B cell subpopulations from infancy to adulthood. *Clin. Exp. Immunol.* **162**, 271–279 (2010).
6. Danaher, P. *et al.* Gene expression markers of Tumor Infiltrating Leukocytes. *J Immunother Cancer* **5**, 18 (2017).
7. Hu, X. *et al.* Landscape of B cell immunity and related immune evasion in human cancers. *Nat. Genet.* **51**, 560–567 (2019).
8. Bolotin, D. A. *et al.* MiXCR: software for comprehensive adaptive immunity profiling. *Nat Methods* **12**, 380–381 (2015).
9. Salcher, S. *et al.* High-resolution single-cell atlas reveals diversity and plasticity of tissue-resident neutrophils in non-small cell lung cancer. *Cancer Cell* **40**, 1503–1520.e8 (2022).
10. Byrska-Bishop, M. *et al.* High-coverage whole-genome sequencing of the expanded 1000 Genomes Project cohort including 602 trios. *Cell* **185**, 3426–3440.e19 (2022).
11. Ryan, J. L. *et al.* Clonal evolution of lymphoblastoid cell lines. *Lab. Invest.* **86**, 1193–1200

(2006).

12. Vettermann, C. & Schlissel, M. S. Allelic exclusion of immunoglobulin genes: models and mechanisms. *Immunol. Rev.* **237**, 22 (2010).

13. Lappalainen, T. *et al.* Transcriptome and genome sequencing uncovers functional variation in humans. *Nature* **501**, 506–511 (2013).

14. Yamanaka, K. *et al.* Unusual feature of the T-cell receptor genes in T-lineage acute lymphoblastic leukemia. *Leuk. Res.* **21**, 667–674 (1997).

15. Meleshko, A. N., Belevtsev, M. V., Savitskaja, T. V. & Potapnev, M. P. The incidence of T-cell receptor gene rearrangements in childhood B-lineage acute lymphoblastic leukemia is related to immunophenotype and fusion oncogene expression. *Leuk. Res.* **30**, (2006).

16. Laskar, R. S. *et al.* Sexual dimorphism in cancer: insights from transcriptional signatures in kidney tissue and renal cell carcinoma. *Hum. Mol. Genet.* **30**, 343–355 (2021).
