# Supplementary material for: ImmuneLENS characterizes systemic immune dysregulation in aging and cancer
Source: Nat Genet. 2025 Feb 18;57(3):694–705. doi: 10.1038/s41588-025-02086-5 (PMC11906351; doi:10.1038/s41588-025-02086-5)
Supplement: Supplementary file 2 — Reporting Summary [file 41588_2025_2086_MOESM2_ESM.pdf]

nature portfolio

Corresponding author(s): Dr Nicholas McGranahan

Last updated by author(s): Nov 5, 2024

Reporting Summary

Nature Portfolio wishes to improve the reproducibility of the work that we publish. This form provides structure for consistency and transparency in reporting. For further information on Nature Portfolio policies, see our Editorial Policies and the Editorial Policy Checklist.

Statistics

For all statistical analyses, confirm that the following items are present in the figure legend, table legend, main text, or Methods section.

n/a	Confirmed	
☐	☒	The exact sample size (*n*) for each experimental group/condition, given as a discrete number and unit of measurement
☐	☒	A statement on whether measurements were taken from distinct samples or whether the same sample was measured repeatedly
☐	☒	The statistical test(s) used AND whether they are one- or two-sided<br>*Only common tests should be described solely by name; describe more complex techniques in the Methods section.*
☐	☒	A description of all covariates tested
☐	☒	A description of any assumptions or corrections, such as tests of normality and adjustment for multiple comparisons
☐	☒	A full description of the statistical parameters including central tendency (e.g. means) or other basic estimates (e.g. regression coefficient) AND variation (e.g. standard deviation) or associated estimates of uncertainty (e.g. confidence intervals)
☐	☒	For null hypothesis testing, the test statistic (e.g. *F*, *t*, *r*) with confidence intervals, effect sizes, degrees of freedom and *P* value noted<br>*Give P values as exact values whenever suitable.*
☒	☐	For Bayesian analysis, information on the choice of priors and Markov chain Monte Carlo settings
☒	☐	For hierarchical and complex designs, identification of the appropriate level for tests and full reporting of outcomes
☐	☒	Estimates of effect sizes (e.g. Cohen's *d*, Pearson's *r*), indicating how they were calculated

Our web collection on statistics for biologists contains articles on many of the points above.

Software and code

Policy information about availability of computer code

Data collection

No software was used to collect data

Data analysis

R version 4.0.2
samtools (1.3.1)
PLINK (1.9)

R packages used:
tidyverse (1.3.2)
ggplot2 (3.4.1)
dplyr (1.1.0)
tidyr (1.3.0)
ggpubr (0.6.0)
scales (1.2.1)
rstatix (0.7.2)
lubridate (1.9.2)
survminer (0.4.9)
survival (3.1-12)
survcomp (1.40.0)
RColorBrewer (1.1-3)
gridExtra (2.3)
gtable (0.3.2)

GGgally (2.1.2)
ggforce (0.4.1)
TCellExTRECT (1.0.1)
MatchIt (4.5.0)
dndscv (0.0.1.0)

The code to estimate T and B cell fractions, B cell class switching and diversity metrics was produced using the custom made ImmuneLENS R package which is available at https://github.com/McGranahanLab/ImmuneLENS.

For manuscripts utilizing custom algorithms or software that are central to the research but not yet described in published literature, software must be made available to editors and reviewers. We strongly encourage code deposition in a community repository (e.g. GitHub). See the Nature Portfolio guidelines for submitting code & software for further information.

Data

Policy information about availability of data

All manuscripts must include a data availability statement. This statement should provide the following information, where applicable:

- Accession codes, unique identifiers, or web links for publicly available datasets
- A description of any restrictions on data availability
- For clinical datasets or third party data, please ensure that the statement adheres to our policy

TRACERx100:
The RNA sequencing (RNA-seq) and whole exome sequencing (WES) data (in each case from the TRACERx study) used during this study is a subset of the TRACERx421 data set and have been deposited at the European Genome–phenome Archive (EGA), which is hosted by The European Bioinformatics Institute (EBI) and the Centre for Genomic Regulation (CRG) under the accession codes EGAS00001006517 (RNAseq), EGAS00001006494 (WES); access is controlled by the TRACERx data access committee. Details on how to apply for access are available at the linked pages. For TCRseq data used in this analysis the FASTQ data is deposited at the Short Read Archive (SRA) under accession code BioProject: PRJNA544699

Coverage files for the TCRA, TCRB, TCRG and IGH loci were generated from WGS TRACERx100 samples. These coverage files used for the calculation of the T and B cell fractions is available at zenodo.org (10.5281/zenodo.7785803) and were the only data derived from the TRACERx WGS analysis used within this paper.

100KGP:
WGS and phenotypic data from the 100,000 Genomes Project can be accessed by application to Genomics England following the procedure outlined at https://www.genomicsengland.co.uk/about-gecip/joining-research-community/.

1000 Genomes:
The 1000 Genome Data used is publicly available and can be accessed on https://www.internationalgenome.org/data. Calculated ImmuneLENS output including class switching and polyclonal predictions for each LCL cell line included are available on zendodo (10.5281/zenodo.1109397).
PCAWG:
PCAWG data used in this study was obtained through our collaboration with the MD Anderson. To gain access to the TCGA portion of the PCAWG data used in this study, researchers need to apply to the TCGA data access committee via dbGaP (https://dbgap.ncbi.nlm.nih.gov/aa/wga.cgi?page=login). The calculated ImmuneLENS output for these samples is available on zenodo.org (10.5281/zenodo.1109396)

TCGA:
To access TCGA WES and low pass WGS data, researchers will need to apply to the TCGA Data Access Committee (DAC) via dbGaP (https://dbgap.ncbi.nlm.nih.gov/aa/wga.cgi?page=login).

The calculated T cell ExTRECT TCRA T cell fraction scores along with the ImmuneLENS output for the low pass WGS samples used in this study is available at zenodo.org (10.5281/zenodo.7794867).
scRNA datasets:
All data used in this analysis is described in Salcher et al50. available at https://cellxgene.cziscience.com/collections/edb893ee-4066-4128-9aec-5eb2b03f8287.

Research involving human participants, their data, or biological material

Policy information about studies with human participants or human data. See also policy information about sex, gender (identity/presentation), and sexual orientation and race, ethnicity and racism.

Reporting on sex and gender	100KGP pan cancer cohort: Breakdown by phenotypic sex classification at birth as provided in the Genomics England Research Environment: Females (8197), Males (6304)<br><br>100KGP healthy cohort: Breakdown by phenotypic sex classification at birth as provided in the Genomics England Research Environment: Females (17169), Males (13496)<br><br>TRACERx: There were 68 male and 32 female non-small cell lung cancer patients in the TRACERx study<br><br>PCAWG: The subset of the PCAWG data used for the analysis contains 308 females and 231 males
Reporting on race, ethnicity, or other socially relevant groupings	Most probable ancestry within the 100KGP is provided in the Genomics England Research Environment based on five broad super-populations (see https://re-docs.genomicsengland.co.uk/ancestry_inference/) for African, Admixed American, East Asian, European and South Asian. An unassigned ancestry group was also used for participants with admixed ancestry where no probability for an individual super-population was above 80%.

The breakdown by ancestry within the 100KGP is as follows:
Healthy cohort:
African (664), Admixed American (99), East Asian (185), European (23636), South Asian (3542), Unassigned (2265), Unknown (274)
Pan cancer cohort:
African (447), Admixed American (32), East Asian (114), European (12489), South Asian (515), Unassigned (577), Unknown (327)

Population characteristics

100KGP:
Healthy cohort consists of 30,665 participants originating from the rare disease cohort arm of the 100KGP and representing the non-affected relatives.
Age breakdown (years): < 20 (1445), 20-24 (537), 25-29 (1807), 30-34 (3942), 35-39 (5465), 40-44 (4787), 45-49 (3954), 50-54 (2745), 55-59 (1798), 60-64 (1295), 65-69 (1010), 70-74 (893), 75-79 (518) ,>80 (469)

Pan cancer cohort consists of 14,501 participants covering 33 main cancer types with samples derived from both tumour tissue and matched germline.
Age breakdown (years): < 20 (284), 20-24 (75), 25-29 (147), 30-34 (194), 35-39 (285), 40-44 (410), 45-49 (780), 50-54 (1129), 55-59 (1446), 60-64 (1790), 65-69 (2154), 70-74 (2208), 75-79 (1569),>80 (1399)

TRACERx:
There were 68 male and 32 female non-small cell lung cancer patients in the TRACERx study, with a median age of 68. The cohort is predominantly early-stage: Ia(26), Ib(36), IIa(13), IIb(11), IIIa(13), IIIb(1). Seventy-two had no adjuvant treatment and
28 had adjuvant therapy.
Patients were recruited into TRACERx according to the following eligibility criteria (taken from the study protocol).
Inclusion criteria:
-Written Informed consent
-Patients ≥18 years of age, with early stage I-IIIA disease who are eligible for primary surgery
-Histopathologically confirmed NSCLC, or a strong suspicion of cancer on lung imaging necessitating surgery (e.g. diagnosis determined from frozen section in theatre)
-Primary surgery in keeping with NICE guidelines planned (see section 9.3)
-Agreement to be followed up in a specialist centre
-Performance status 0 or 1
-Suspected tumour at least 15mm in diameter on pre-operative imaging
Exclusion criteria:
-Any other current malignancy or malignancy diagnosed or relapsed within the past 5 years (other than non-melanomatous skin
cancer, stage 0 melanoma in situ, and in situ cervical cancer)
-Psychological condition that would preclude informed consent
-Treatment with neo-adjuvant therapy for current lung malignancy deemed necessary
-Adjuvant therapy other than platinum-based chemotherapy and/or radiotherapy
-Known Human Immunodeficiency Virus (HIV), Hepatitis B Virus (HBV), Hepatitis C Virus (HCV) or syphilis infection.
-Sufficient tissue, i.e. a minimum of two tumour regions, is unlikely to be obtained for the study based on pre-operative imaging
Patient ineligibility following registration:
-There is insufficient tissue
-The patient is unable to comply with protocol requirements
-There is a change in histology from NSCLC following surgery, or NSCLC is not confirmed during or after surgery.
-Change in staging to IIIB/IV following surgery
-The operative criteria are not met (e.g. incomplete resection with macroscopic residual tumours (R2)); see section 9.3 for a list
of accepted surgical procedures. Patients with microscopic residual tumours (R1) are eligible and should remain in the study
-Adjuvant therapy other than platinum-based chemotherapy and/or radiotherapy is administered.

Recruitment

100KGP: Cases were recruited by referring clinicians through the National Health Service

TRACERx:
Patients seen with a new diagnosis of lung cancer in lung cancer units across the United Kingdom, according to the eligibility criteria above, were recruited. No selection bias has been identified to date.
All patient tumor regions with RIN scores > 5 were used for RNA-sequencing and analyzed in this study.
All patients were assigned a study ID that was known to the patient. These were subsequently converted to linked study Ids such
that the patients could not identify themselves in study publications. All human samples, tissue and blood, were linked to the study ID and barcoded such that they were anonymised and tracked on a centralised database overseen by the study sponsor only.
Informed consent for entry into the TRACERx study was mandatory and obtained from every patient.

PCAWG:
Patients were recruited by the participating centres following local protocols. Samples obtained had to meet criteria on amount of tumour DNA available, meaning that the cohort is potentially somewhat biased towards larger tumours. Otherwise, we anticipate no major recruitment biases.

Ethics oversight

100KGP:
The 100,000 Genomes project was approved by East of England–Cambridge Central Research Ethics Committee ref:20/

EE/0035.
TRACERx:
The TRACERx study (Clinicaltrials.gov no: NCT01888601) is sponsored by University College London (UCL/12/0279) and has been approved by an independent Research Ethics Committee (13/LO/1546)
PCAWG: The Ethics oversight for the PCAWG protocol was undertaken by the TCGA Program Office and the Ethics and Governance Committee of the ICGC. Each individual ICGC and TCGA project that contributed data to PCAWG had their own local
arrangements for ethics oversight and regulatory alignment.

Note that full information on the approval of the study protocol must also be provided in the manuscript.

Field-specific reporting

Please select the one below that is the best fit for your research. If you are not sure, read the appropriate sections before making your selection.

☒ Life sciences ☐ Behavioural & social sciences ☐ Ecological, evolutionary & environmental sciences

For a reference copy of the document with all sections, see nature.com/documents/nr-reporting-summary-flat.pdf

Life sciences study design

All studies must disclose on these points even when the disclosure is negative.

Sample size	No sample size calculations were performed, we analysed existing datasets, namely the TRACERx100 cohort and the 100KGP cohort.<br><br>The TRACERx100 data set was chosen as it was an existing cohort containing orthogonal immune data (RNAseq, TCRseq) for which ImmuneLENS could be easily validated. The 100KGP dataset was chosen due to being a large WGS cohort without any orthogonal immune related data for which insights on regulation of circulating and infiltrating immune cell fractions could be gained.<br><br>Additional validation was done on:<br>1) 1000 genome cohort, which was chosen due to containing WGS samples originating from B cell derived cell lines to specifically validate our B cell fractions.<br>2) PCAWG and TCGA cohorts data sets for validation of our pan cancer analysis results from the 100KGP on a separate data set.
Data exclusions	Within the 100KGP pan cancer cohort participants with multiple tumour samples were excluded due to lack of annotation of the reason for multiple samples (e.g. technical resequencing, representative of metastasis, multiple region sequenced or occurrence of a second primary tumour at a later time point) these were removed from the pan cancer cohort. All germline samples not derived from blood samples were also excluded from the analysis due to our focus on circulating immune fraction in this study.
Replication	This study was on pre-existing data sets and hence findings were not replicated
Randomization	No randomization or permutation analysis was performed in this study, samples were split based on either categorical data or threshold values e.g. for the TCRA T cell fraction.
Blinding	Blinding was not applicable in this study, all data was from pre-existing data and there was no control and treatment arms involved

Reporting for specific materials, systems and methods

We require information from authors about some types of materials, experimental systems and methods used in many studies. Here, indicate whether each material, system or method listed is relevant to your study. If you are not sure if a list item applies to your research, read the appropriate section before selecting a response.

Materials & experimental systems

n/a	Involved in the study	
☒	☐	Antibodies
☒	☐	Eukaryotic cell lines
☒	☐	Palaeontology and archaeology
☒	☐	Animals and other organisms
☐	☒	Clinical data
☒	☐	Dual use research of concern
☒	☐	Plants

Methods

n/a	Involved in the study	
☒	☐	ChIP-seq
☒	☐	Flow cytometry
☒	☐	MRI-based neuroimaging

Clinical data

Policy information about clinical studies

All manuscripts should comply with the ICMJE guidelines for publication of clinical research and a completed CONSORT checklist must be included with all submissions.

Clinical trial registration	TRACERx100: NCT01888601<br>100KGP: N/A
Study protocol	TRACERx100: The study protocol is available at NEJM.org linked to Jamal-Hanjani et al NEJM 2017 (PMID: 28445112)<br>100KGP: Refer to Genomic England Limited website for information on data collection.
Data collection	TRACERx100: Patients seen with a new diagnosis of lung cancer in lung cancer units across the United Kingdom, according to the eligibility<br>criteria outlined in the study protocol, were recruited. No selection bias has been identified to date.<br>All patient tumor regions with RIN scores > 5 were used for RNA-sequencing and analyzed in this study.<br>All patients were assigned a study ID that was known to the patient. These were subsequently converted to linked study Ids such that the patients could not identify themselves in study publications. All human samples, tissue and blood, were linked to the study ID and barcoded such that they were anonymised and tracked on a centralised database overseen by the study sponsor only.<br>Informed consent for entry into the TRACERx study was mandatory and obtained from every patient<br>100KGP: Refer to Genomic England Limited website for information on data collection.
Outcomes	TRACERx100: The outcome measures of the TRACERx trial are intratumour heterogeneity, disease-free survival, and overall survival.<br>100KGP: N/A
